# Narrative worlds as developmental environments: a Narrative Ecological Framework for understanding story learning and interpretation

**DOI:** 10.3389/fpsyg.2026.1934346

**Published:** 2026-10-07

**Authors:** Jagdeep Panchal, Ganesh Chilkhe, Divya Muraleedharan, Suseela Maddirala, Praveen Gudidinni

**Affiliations:** 1 Koneru Lakshmaiah Education Foundation, KL Deemed to be University, Hyderabad, Telangana, India; 2 School of Social Sciences and Languages, Vellore Institute of Technology, Chennai, Chennai, India; 3 Rockwell Business School of Management, Hyderabad, Telangana, India

**Keywords:** Narrative Ecological Framework, Narrative Ecological Configuration, story learning, children’s literature, Pattern Extraction, Pattern Abstraction, interpretive orientations

## Abstract

Stories provide children with recurring encounters with characters, relationships, actions, situations, outcomes, and representations of social and experiential worlds. Research on narrative comprehension, story schemas, empathy, social cognition, and related forms of learning has documented important functions of narrative engagement, but less attention has been given to how the organisation of narrative worlds as a whole may contribute to recurring patterns of interpretation. This paper introduces the Narrative Ecological Framework (NEF), a conceptual framework for examining narrative worlds as organised environments in which multiple dimensions interact. NEF identifies six analytically distinguishable but interconnected dimensions: Agency Organisation, Relational Organisation, Practice Organisation, Situational Organisation, Outcome Organisation, and Representation Organisation. Their interaction produces a Narrative Ecological Configuration (NEC), providing an analytical basis for describing and comparing how narrative worlds organise action, relationships, practices, situations, outcomes, and their representation. The framework further proposes that when similar NECs recur across narrative experiences, readers may encounter recurring organisational regularities that can potentially become available for interpretation. Pattern Extraction and Pattern Abstraction are proposed as possible cognitive processes through which such regularities may be recognised and represented at a more general level, while interpretive orientations are conceptualised as possible tendencies through which recurring configurations may become relevant to subsequent interpretation. NEF does not posit a deterministic relationship between narrative configurations and particular beliefs, values, or interpretations; rather, it proposes a conceptual pathway that remains open to empirical investigation. The framework also distinguishes NEC as a narrative-level construct from Configurational Understanding as a reader-level construct, providing a basis for future research examining whether the capacity to recognise and integrate interactions among narrative dimensions is associated with deeper narrative comprehension. The paper concludes by outlining potential educational applications, limitations, and directions for empirical testing.

## 1 Introduction

Children’s stories occupy an important place in educational, cultural, and everyday experiences, providing young readers with opportunities to encounter characters, relationships, actions, problems, and consequences in meaningful narrative contexts. Through folktales, fables, myths, literary narratives, and classroom texts, children encounter representations of social interactions and situations that extend beyond the immediate circumstances of their own lives. Consequently, research on children’s narratives has examined their contributions to comprehension, literacy development, empathy, moral reasoning, and social understanding (Mar, 2018; Schrijvers et al., 2019; Coats, 2020). Such work establishes the importance of stories in children’s cognitive, linguistic, and social development, while also suggesting that the significance of narrative engagement may extend beyond the acquisition of particular skills or the understanding of individual story elements.

Existing research has often examined specific narrative functions or individual features, such as comprehension, vocabulary, empathy, moral learning, and social cognition, providing valuable accounts of particular dimensions of story learning but a more limited account of how the organisation of the narrative world as a whole may contribute to recurring patterns of interpretation. A narrative does not present characters, relationships, actions, problems, and outcomes as isolated components; rather, these elements are organised in relation to one another to construct a particular world of action, interaction, practice, consequence, and meaning and may thereby make particular assumptions about social relations and possibilities available for interpretation (Stephens, 1992). Understanding how such recurring configurations may become available to young readers therefore requires attention not only to what narrative elements are present, but also to how they are organised and interact within the narrative world.

Narrative worlds can therefore be understood as more than settings in which events occur. They are constructed through the organisation of characters, their goals and actions, their relationships with others, the practices through which they respond to circumstances, and the consequences that follow. Narrative comprehension requires readers to integrate events, character goals, actions, causal connections, and background knowledge into a coherent representation of the narrative (Lorch and Hayden, 2020). Such comprehension may also involve interpreting characters’ mental states, intentions, and perspectives, a concern examined in cognitive approaches to fiction (Zunshine, 2006). Research with children also demonstrates that the narrative context in which linguistic information is presented can influence how that information is interpreted, with narrative settings facilitating attention to characters’ perspectives in the interpretation of reported speech (Köder and Maier, 2018).

This perspective shifts attention from stories as collections of discrete narrative elements to narrative worlds as organised environments in which patterns of action, interaction, knowledge, circumstance, and consequence are made available to readers (Herman, 2009). Narrative worlds are not simply backdrops for events; they provide structured contexts in which characters act, relationships are negotiated, practices are enacted, situations emerge, and outcomes acquire significance. Research on young children’s narrative activity likewise suggests that stories and narrative play provide contexts in which children construct and explore different kinds of fictional worlds, with actions, objects, and people construed in particular ways (Engel, 2005). These perspectives suggest that the developmental significance of stories may lie partly in the configurational patterns through which narrative worlds organise what characters do, how they relate, how they respond to circumstances, and what follows from those responses. Building on this perspective, the present paper proposes that recurring configurations within narrative worlds may provide interpretive conditions through which young readers encounter and develop broader interpretive orientations.

The six dimensions concern, respectively, the organisation of agency, relationships, practices, situations, outcomes, and representation within narrative worlds. The dimensions are not intended as a fixed sequence or deterministic causal chain. Rather, they provide interconnected analytical lenses through which the organisation of a narrative world can be examined.

The interaction of these dimensions gives rise to what this paper terms a Narrative Ecological Configuration (NEC): a particular pattern in which agency, relationships, practices, situations, outcomes, and representation are organised within a narrative world. An NEC is therefore not an additional narrative element or a fixed type of story, but an emergent configuration produced through the varying prominence and interaction of the six dimensions. Two narratives may contain similar characters, events, or themes while nevertheless constructing different ecological configurations because the relationships among their dimensions differ. This configurational perspective builds on the broader understanding of narrative worlds as constructed and dynamically organised environments (Herman, 2009), while extending that perspective toward the developmental question of what kinds of recurring organisational patterns young readers may encounter across stories. NEF consequently directs attention from isolated narrative features to the patterns generated through their interaction.

The significance of these configurations, however, depends not only on how narrative worlds are organised but also on how readers engage with and make sense of that organisation. Narrative comprehension involves constructing a representation of the storyworld by integrating characters, events, goals, actions, causal relations, and other contextual information, while readers’ existing knowledge contributes to the interpretation of narratively organised information (Lorch and Hayden, 2020; Paris and Paris, 2003). Research on story schema has further shown that children’s comprehension of narratives involves structured representations of recurring story components and their relationships (Stein and Glenn, 1979). Building on this cognitive perspective, NEF proposes that readers may also encounter and gradually recognise broader configurational patterns across narrative experiences. Such recognition need not imply conscious identification of the six dimensions as formal categories; rather, it refers to the possibility that recurring configurations become available as patterns through which narrative situations and relationships are interpreted.

The present paper develops NEF as a conceptual account of how narrative worlds may function as developmental environments for young readers (Herman, 2009; Engel, 2005). It does not seek to establish causal or predictive relationships between particular narrative configurations and particular interpretive outcomes. Instead, it establishes the six-dimensional architecture of narrative-world organisation, defines Narrative Ecological Configuration, and proposes how recurring configurations may become relevant to young readers’ interpretive orientations. The framework also distinguishes the organisation of the narrative world from readers’ configurational understanding of that organisation, providing a basis for subsequent empirical investigation into whether learners who more effectively recognise and integrate interactions among the six dimensions demonstrate deeper narrative comprehension. By shifting attention from isolated narrative elements to their interconnected organisation, NEF provides a framework for examining stories not only as texts to be understood but also as structured environments through which young readers may encounter recurring ways of organising action, relationships, knowledge, situations, outcomes, and meaning.

## 2 Conceptual and theoretical background

The idea that narratives can influence learning and interpretation rests on several complementary lines of inquiry. Narrative theory has examined how stories construct or invite readers to construct worlds populated by particular agents, events, places, temporal relations, and experiential perspectives (Herman, 2009; Ryan, 1991). Cognitive research has further shown that readers do not simply register these elements independently but integrate them into coherent representations of narrative situations and events (Lorch and Hayden, 2020; Paris and Paris, 2003). Research on story schemas similarly suggests that readers develop knowledge of recurring narrative structures and relationships among story components (Mandler and Johnson, 1977; Stein and Glenn, 1979). Together, these perspectives provide a basis for considering narrative engagement as an organised process of meaning construction rather than the processing of isolated textual features. The present framework builds on this foundation but shifts the analytical focus towards the organisation of the narrative world itself and the possible significance of recurring configurations of that organisation.

### 2.1 Narrative worlds and worldmaking

Narrative scholarship has increasingly drawn attention to the worlds that narratives construct or invite readers to construct, moving beyond an understanding of stories as sequences of events or collections of discrete textual elements. The concept of the storyworld foregrounds the situated environments in which narrative events, characters, actions, and experiences become meaningful. Herman (2009), for example, describes narratives as blueprints for worldmaking and examines how readers configure storyworlds through dimensions of what, where, and when. Similarly, possible-worlds approaches to narrative emphasise the construction of alternative or imagined worlds and the relations among the entities, states, and events that constitute them (Ryan, 1991). These perspectives establish the narrative world as an important level of analysis in which individual narrative components acquire meaning through their placement within a larger configuration. Unlike plot, which concerns the organisation and progression of events, or theme, which concerns broader meanings or ideas derived from those events, the narrative world concerns the organised conditions within which characters act, relationships develop, situations emerge, and outcomes acquire significance (Bal, 2017; Herman, 2009).

The concept of narrative world is also relevant to how readers construct meaning from stories. Narrative understanding requires readers to build coherent representations of characters, events, actions, goals, temporal and causal relations, and the situations in which these elements unfold (Lorch and Hayden, 2020; Paris and Paris, 2003). From this perspective, comprehension is not simply the recognition of separate narrative details but involves integrating information into an organised representation of the storyworld. Such organisation is particularly relevant to children’s engagement with stories because young readers encounter characters, actions, relationships, problems, and consequences as interconnected components of narrative situations. Research on children’s narrative understanding and story schemas likewise indicates that readers develop knowledge of recurring structures and relations among story components (Mandler and Johnson, 1977; Stein and Glenn, 1979).

These perspectives provide an important foundation for the present framework, but they do not by themselves specify how the internal organisation of a narrative world can be examined as an integrated environment for experience and interpretation. Developmental perspectives emphasise that development occurs through interactions across multiple contexts, including social, educational, and experiential environments (Bronfenbrenner, 1979; Bronfenbrenner and Morris, 2006). In this broader sense, narrative worlds may be considered potential developmental environments because readers repeatedly encounter organised representations of actions, relationships, situations, and consequences within them. The Narrative Ecological Framework (NEF) brings these lines of inquiry together by shifting attention towards how agency, relationships, practices, situations, outcomes, and representation are organised and interact within a narrative world. The purpose is not to replace existing accounts of storyworld construction or narrative comprehension, but to provide an additional analytical level for examining the recurring configurations through which narrative worlds may become relevant to young readers’ interpretation.

In NEF, the term “ecological” refers specifically to the relational organisation and interaction of conditions within a narrative world rather than to physical ecology or to an equivalence between fictional environments and the real-world developmental systems conceptualised by Bronfenbrenner (1979). The framework draws from ecological developmental theory the broader principle that experiences can be understood in relation to organised and interacting contextual conditions, but it does not map narrative worlds onto Bronfenbrenner’s (1979) microsystem, mesosystem, exosystem, or macrosystem. Instead, NEF extends this contextual and relational perspective to the analysis of fictional or represented environments by examining how agency, relationships, practices, situations, outcomes, and representation are organised together within a narrative world. The ecological designation therefore identifies the analytical level and relational logic of the framework, while the developmental consequences of readers’ encounters with such environments remain propositions requiring empirical investigation.

### 2.2 Narrative comprehension and the integration of narrative information

Narrative comprehension involves more than identifying individual characters, events, or textual details; readers construct an integrated representation of the situations and events that constitute the storyworld by connecting characters with their goals, actions, events, consequences, and causal relations while incorporating relevant background knowledge (Kendeou et al., 2016; Lorch and Hayden, 2020). Such integration is particularly important for understanding why events occur and how one development relates to another within a story. From a cognitive perspective, therefore, comprehension involves constructing relations among narrative components rather than merely accumulating information about them.

Research on children’s story comprehension further indicates that young readers develop knowledge of recurring organisational patterns within narratives. Classic work on story structure identified regularities in how events and episodes are organised and recalled (Mandler and Johnson, 1977), while Stein and Glenn (1979) demonstrated the importance of structured story representations in children’s comprehension. These perspectives suggest that readers can move beyond individual narrative instances to recognise recurring relationships among characters, goals, actions, problems, and outcomes. Such recognition provides an important cognitive foundation for considering how repeated exposure to organised narrative environments might make broader patterns available for subsequent interpretation.

For NEF, however, the relevant unit is broader than conventional story structure. The framework is concerned not only with whether a narrative contains recognisable episodes or components, but with how multiple dimensions of the narrative world are organised in relation to one another. Thus, a learner may understand that a character encounters a problem and eventually resolves it while also encountering a broader configuration concerning who possesses agency, how relationships influence action, how knowledge and decisions inform practices, how situations develop, and how outcomes are represented. This distinction provides the cognitive bridge between narrative-world organisation and the possibility of configurational understanding.

### 2.3 Story schemas and recurring narrative structures

Research on story schemas provides an important cognitive basis for understanding how readers may recognise regularities across narrative experiences (Mandler and Johnson, 1977; Stein and Glenn, 1979). Classic accounts of story structure proposed that narratives are organised through recurring relations among events and episodes, enabling readers to develop structured expectations about how stories are organised and how their components relate to one another (Mandler and Johnson, 1977; Stein and Glenn, 1979). Research on children’s narrative comprehension likewise emphasises the integration of narrative information into coherent representations (Paris and Paris, 2003; Lorch and Hayden, 2020). Taken together, these perspectives provide a basis for considering how recurring organisational patterns across narratives may become available as resources for understanding subsequent stories.

For the present framework, however, recurring narrative structure is broader than conventional story grammar or episodic organisation. The relevant question is not only whether readers recognise familiar sequences of events, but whether they may also become sensitive to recurring patterns in how narrative worlds organise agency, relationships, practices, situations, outcomes, and representation. This distinction provides an important conceptual foundation for NEF. Rather than assuming that readers consciously identify these dimensions as formal categories, the framework considers whether their recurring organisation across narrative experiences may become cognitively meaningful and potentially relevant to subsequent interpretation.

## 3 Narrative Ecological Framework

The preceding discussion establishes that narratives construct organised storyworlds, that readers integrate narrative information into coherent representations, and that recurring narrative structures may become cognitively available across experiences. Building on these foundations, the Narrative Ecological Framework (NEF) conceptualises the narrative world as an organised developmental environment whose characteristics cannot be adequately captured through isolated analysis of plot, character, theme, or moral lesson. Instead, NEF focuses on the ways in which multiple dimensions of narrative-world organisation operate together to construct the conditions within which characters act, relate, make decisions, encounter situations, experience consequences, and interpret what follows. The framework identifies six analytically distinguishable but functionally interconnected dimensions: Agency Organisation, Relational Organisation, Practice Organisation, Situational Organisation, Outcome Organisation, and Representation Organisation. These dimensions are not proposed as sequential stages or deterministic causal components. Rather, they provide complementary analytical lenses for examining how a narrative world is organised and how different dimensions may reinforce, modify, or constrain one another.

### 3.1 Six dimensions of narrative-world organisation

The Narrative Ecological Framework conceptualises the organisation of a narrative world through six analytically distinguishable but functionally interconnected dimensions: Agency Organisation, Relational Organisation, Practice Organisation, Situational Organisation, Outcome Organisation, and Representation Organisation. Together, these dimensions describe how a narrative constructs its agents, organises their relationships, translates those characteristics and relationships into practices, generates and develops situations through those practices, addresses the resulting situations, and represents what has occurred and what it means within the continuing narrative world. The dimensions therefore provide complementary analytical lenses for examining the organisation of a narrative world rather than treating characters, actions, events, and outcomes as isolated elements.

Figure 1 provides an overview of how these six dimensions fit within the broader architecture of NEF, including their integrated organisation as a Narrative Ecological Configuration (NEC), the proposed significance of recurring NECs across narrative experiences, and the reader-level construct of Configurational Understanding.

**Figure 1:**
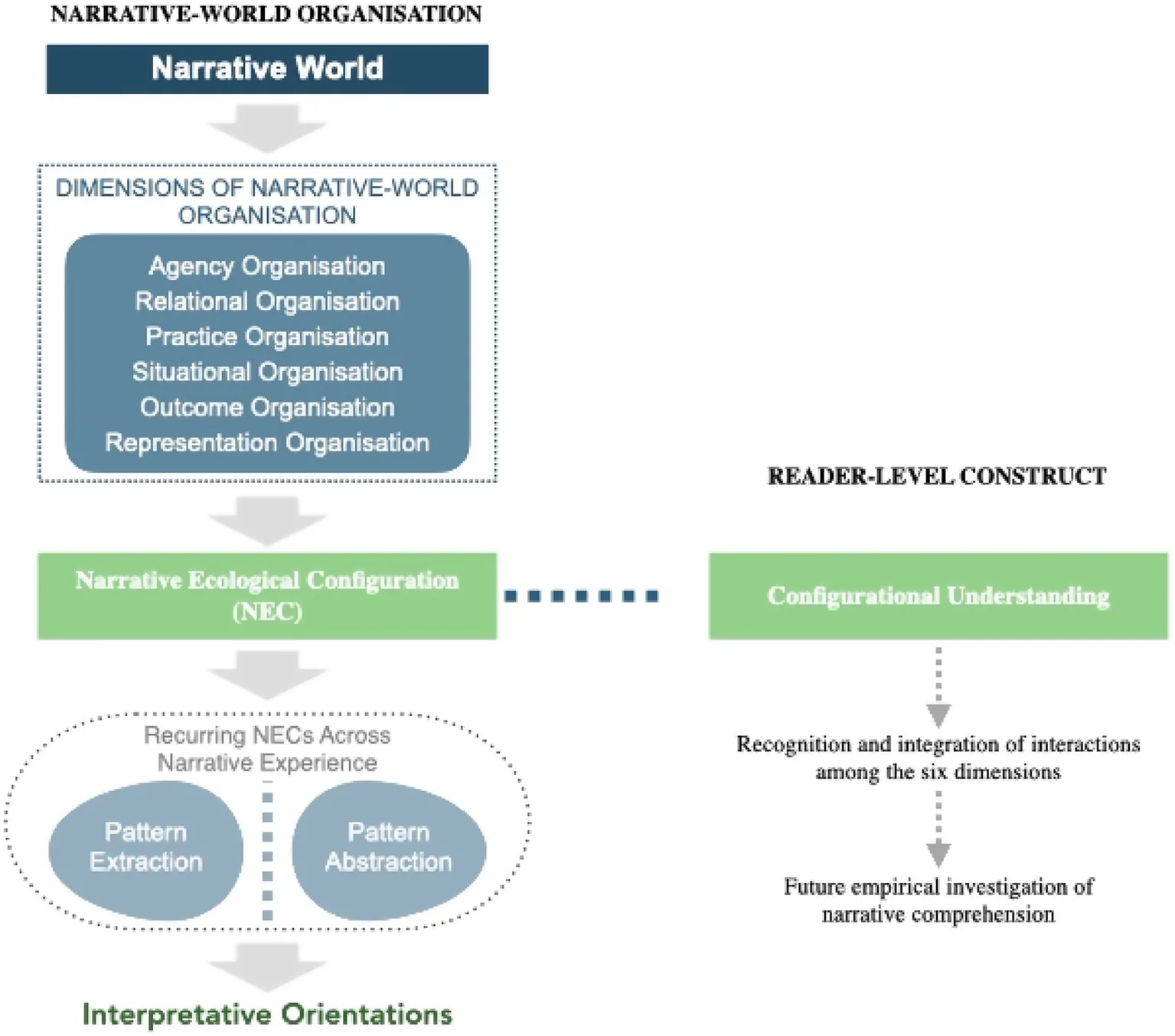
Conceptual architecture of the Narrative Ecological Framework. The figure distinguishes narrative-world organisation from the reader-level construct of Configurational Understanding. The six dimensions are integrated within a Narrative Ecological Configuration (NEC); recurring NECs may be relevant to Pattern Extraction, Pattern Abstraction, and interpretive orientations. Configurational Understanding refers to the reader’s recognition and integration of interactions among the six dimensions and provides a basis for future empirical investigation of narrative comprehension. Connectors indicate proposed relationships rather than deterministic or established causal pathways.

The six dimensions are proposed as a provisional analytical architecture based on three related considerations. First, each dimension addresses a distinguishable aspect of narrative-world organisation: the positioning of agents, the organisation of relationships, the enactment of capacities and relationships through practices, the emergence and development of situations, the addressing and consequences of those situations, and the representation of these developments. Second, the dimensions are functionally complementary: each captures an aspect of narrative organisation that is analytically separable but connected to the others within the developing narrative world. Third, their combined use permits examination of configurations that may not be visible when these aspects are considered independently. The six dimensions are therefore not intended as an exhaustive taxonomy of all possible properties of narrative worlds, but as a theoretically specified analytical starting point whose adequacy and potential refinement remain open to comparative and empirical investigation.

The six dimensions are proposed as analytical dimensions that may be applied across cultural and narrative contexts, rather than as claims that agency, relationships, practices, situations, outcomes, or representation have identical meanings or normative significance across cultures. Their manifestation, salience, and interpretation may vary according to cultural, historical, and narrative contexts. The framework therefore treats the dimensions as theoretically informed analytical lenses rather than as objective or culture-neutral properties of narrative worlds; their conceptual adequacy and cross-cultural applicability remain open to comparative investigation.

To make the analytical scope of the six dimensions explicit, Table 1 summarises the central question and primary focus associated with each dimension. The table is intended as an analytical guide rather than as a set of independent narrative components; the dimensions are examined in relation to one another within particular narrative worlds.

**Table 1:** Six dimensions of narrative-world organisation in the Narrative Ecological Framework.

Dimension	Central analytical question	Primary analytical focus
Agency Organisation	How are characters positioned as agents capable of acting, influencing, or responding?	The construction and positioning of characters through their capacities, motivations, choices, opportunities, constraints, influence, and responses within the narrative world.
Relational Organisation	How are characters and other relevant story-world entities positioned in relation to one another?	The construction, maintenance, negotiation, and transformation of relationships, including cooperation, dependence, care, trust, support, authority, opposition, competition, conflict, negotiation, affiliation, and exclusion.
Practice Organisation	How are characters’ capacities, relationships, knowledge, decisions, and intentions enacted through purposeful activity?	Actions, decisions, knowledge use, learning, reasoning, problem-solving, planning, cooperation, negotiation, care, assistance, resistance, and treatment of others through which agency and relationships are enacted.
Situational Organisation	What situations emerge, develop, change, or become consequential?	Problems, conflicts, incidents, challenges, discoveries, opportunities, disruptions, changing circumstances, and other developments that characters encounter, generate, or transform and that alter possibilities for subsequent action.
Outcome Organisation	How are situations addressed, transformed, resolved, continued, or otherwise carried forward, and what follows from them?	Resolution, transformation, management, continuation, consequence, recognition, loss, change, and the emergence of subsequent developments, including situations that remain unresolved or produce competing outcomes.
Representation Organisation	How are characters, relationships, practices, situations, and outcomes represented, framed, evaluated, emphasised, and carried forward?	Foregrounding and backgrounding, attribution of significance, perspective, framing, emphasis, evaluation, connection, visibility, and the ways narrative representation reinforces, complicates, qualifies, challenges, or carries forward meaning.

The framework does not assume that all six dimensions will be equally prominent in every narrative. A dimension may be strongly instantiated, weakly instantiated, or of limited analytical salience in a particular narrative. Such variation should be recorded as part of the analysis rather than treated as a deficiency or as a reason to force textual evidence into a dimension that is not meaningfully supported.

To facilitate the systematic application of these six dimensions, an illustrative analytical matrix is provided in Appendix A. The matrix demonstrates one possible approach to examining narrative worlds through the six dimensions and provides a practical basis for identifying and describing Narrative Ecological Configurations.

The six dimensions provide complementary perspectives on different aspects of narrative-world organisation, but their analytical value lies in their interconnection. The following subsections clarify the scope of each dimension before their integrated organisation is examined through the concept of Narrative Ecological Configuration.

Agency Organisation concerns the construction and positioning of characters as agents with particular capacities, motivations, choices, and opportunities to influence action. Relational Organisation concerns how characters and other relevant story-world entities are positioned in relation to one another. Practice Organisation examines how agency, relationships, knowledge, and intentions are enacted through purposeful activities. Situational Organisation concerns the situations that emerge, develop, or change through these activities. Outcome Organisation examines how such situations are addressed, transformed, resolved, continued, or otherwise carried forward. Finally, Representation Organisation concerns how characters, relationships, practices, situations, and outcomes are framed, emphasised, evaluated, and connected within the narrative.

The six dimensions should not be understood as six sequential stages through which every narrative necessarily progresses. Their ordering provides a functional analytical logic, but their actual operation within a narrative may be recursive, overlapping, and mutually influential. A situation may reshape relationships; an outcome may alter a character’s agency; changes in relationships may generate new practices; and representation may influence how earlier situations or outcomes acquire continuing significance. The analytical value of the framework therefore lies not in isolating six independent dimensions but in examining how they are configured and interact within particular narrative worlds. Their integrated organisation constitutes the basis for identifying a Narrative Ecological Configuration (NEC), developed in the following section.

#### 3.1.1 Agency organisation

Agency Organisation concerns how a narrative constructs and positions characters as agents capable of acting, initiating, deciding, influencing events, responding to circumstances, and pursuing goals. Narratological approaches to character have long considered characters as participants in the storyworld whose attributes, roles, actions, and development contribute to the organisation of narrative events (Garvey, 1978; Margolin, 2007). Discussions of character agency further emphasise characters’ capacity to make choices and exercise influence within the unfolding narrative (Cole, 2018). Building on these perspectives, NEF treats agency not simply as an attribute of an individual character but as an organisational dimension of the narrative world.

Agency Organisation therefore examines whose actions matter, whose decisions initiate or alter developments, whose intentions influence events, whose choices are constrained, and whose responses carry consequences. Agency may be concentrated in a single character, distributed across several characters, shared collectively, constrained by circumstances, or altered during the course of a narrative. A character may therefore possess considerable agency in one situation but limited agency in another. These variations establish conditions within which relationships, practices, situations, and outcomes are organised.

Agency Organisation should nevertheless be distinguished from conventional character analysis. Character analysis may examine traits, motivations, roles, relationships, and development, whereas Agency Organisation asks how those character constructions position characters as capable of producing, influencing, resisting, or responding to change within the narrative world. It should also be distinguished from Practice Organisation. Agency concerns the capacity, positioning, intention, and exercise of choice that make action possible, whereas Practice Organisation concerns how such capacities and relationships become enacted through decisions, knowledge use, learning, problem-solving, treatment of others, and other forms of purposeful activity. Agency Organisation therefore provides one dimension of the narrative configuration without being treated as a determining cause of the other dimensions.

#### 3.1.2 Relational organisation

Relational Organisation concerns how characters and other relevant entities within the narrative world are positioned in relation to one another and how those relationships are constructed, maintained, negotiated, challenged, or transformed. Narrative relationships are not merely background connections among characters; they contribute to how characters function as participants and agents within the storyworld and how their actions and interactions acquire narrative significance (Margolin, 2007). Relational Organisation extends this insight by treating the organisation of relationships as a distinct dimension of the narrative world.

The central analytical question of Relational Organisation is: How are characters and other relevant story-world entities positioned in relation to one another, and what kinds of relationships are constructed through their interactions?

Relational Organisation examines patterns of cooperation, dependence, care, trust, support, authority, opposition, competition, conflict, negotiation, affiliation, exclusion, and other forms of relational positioning. It also considers whether relationships are reciprocal or asymmetrical, stable or changing, individual or collective, and whether they enable, constrain, or transform the actions of those involved. The focus is therefore not simply on identifying who is related to whom, but on examining what kind of relational structure the narrative constructs and how that structure operates within the storyworld.

Relational Organisation should be distinguished from Agency Organisation and Practice Organisation. Agency Organisation concerns how characters are positioned as capable of acting, deciding, initiating, influencing, or responding; Relational Organisation concerns how those agents are positioned in relation to one another. Practice Organisation, in turn, concerns how these capacities and relationships become enacted through actions, decisions, knowledge use, learning, problem-solving, treatment of others, and other purposeful practices. The dimensions may therefore interact closely without being collapsed into one another. A relationship may enable a particular form of agency, while an action may strengthen, challenge, or transform an existing relationship.

#### 3.1.3 Practice organisation

Practice Organisation concerns how characters’ capacities, relationships, knowledge, intentions, and decisions become enacted through purposeful activity within the narrative world. It therefore moves beyond asking what characters are capable of doing or how they are related to examine how those capacities and relationships are expressed in practice. Narrative comprehension research indicates that characters’ goals are closely connected to their actions and the unfolding of narrative events; children’s ability to infer characters’ goals is also related to constructing coherent representations of narratives (Lynch and van den Broek, 2007).

The central analytical question of Practice Organisation is: How are characters’ capacities, relationships, knowledge, decisions, and intentions enacted through practices within the narrative world?

Practice Organisation includes actions and decisions as well as knowledge use, learning, reasoning, problem-solving, planning, cooperation, negotiation, care, assistance, resistance, and the treatment of others. It therefore encompasses both observable actions and the knowledge-oriented or decision-making practices through which characters respond to circumstances. Knowledge is not treated as a separate dimension because its narrative significance within NEF lies primarily in how it is acquired, interpreted, shared, challenged, applied, or transformed through practice. Similarly, learning is considered insofar as it is enacted through characters’ experiences, decisions, responses, and changing practices.

Practice Organisation should be distinguished from Agency Organisation and Relational Organisation: Agency Organisation concerns how characters are positioned as capable of acting, deciding, initiating, influencing, or responding; Relational Organisation concerns how those agents are positioned in relation to one another; and Practice Organisation concerns how these capacities and relationships become enacted through what characters do, decide, know, learn, and negotiate. The same practice may therefore express agency and relational positioning. For example, a character’s decision to assist another may enact individual agency while simultaneously expressing an existing relationship and potentially transforming that relationship.

Practice Organisation also provides an important bridge to Situational Organisation. Practices do not merely occur within situations; they can contribute to the emergence, transformation, escalation, or resolution of situations. Consequently, the analytical focus is not simply on listing actions but on examining how patterns of practice participate in the organisation of the narrative world.

#### 3.1.4 Situational organisation

Situational Organisation concerns the emergence, development, transformation, and interconnection of the situations within which narrative action unfolds. Narrative theory commonly treats events not simply as isolated actions but as developments that alter states, circumstances, or relations within a storyworld (Herman, 2009; Prince, 1982). A situation may involve a conflict, problem, challenge, incident, disruption, change in circumstances, or other development that creates conditions requiring characters to respond. Narrative events can include actions performed by characters as well as situations, incidents, experiences, and changes affecting characters or their environments; such developments contribute to changes of state within the storyworld.

The central analytical question of Situational Organisation is: What situations emerge, develop, change, or become consequential through the practices of characters and the conditions of the narrative world?

Situational Organisation therefore examines what happens to and around characters, how problems or conflicts arise, how circumstances change, how challenges develop, and how one situation may generate or transform another. It is not restricted to conflict and may include a misunderstanding, discovery, unexpected event, environmental change, social problem, practical challenge, interpersonal disagreement, opportunity, loss, achievement, or other circumstance that alters what characters can or need to do. Situations may be initiated through characters’ practices or arise from circumstances beyond an individual character’s immediate control. Their significance lies in how they create, alter, constrain, or open possibilities for subsequent action.

Situational Organisation should be distinguished from Practice Organisation and Outcome Organisation. Practice Organisation examines how characters enact capacities, relationships, knowledge, decisions, and intentions through purposeful activity; Situational Organisation examines the circumstances and developments that those practices generate, encounter, or transform; and Outcome Organisation then examines what happens after or through the development of those situations, including how problems are addressed, conflicts are resolved or left unresolved, situations are transformed, achievements are recognised, or consequences are carried forward. Conflict resolution is therefore not treated as a separate dimension but as one possible form of situational development and outcome handling within the broader configuration.

Situational Organisation is therefore not equivalent to conventional plot or conflict analysis. Its purpose is to examine how situations are organised within the broader narrative ecology and how they connect characters’ practices with subsequent outcomes. A particular situation may reshape relationships, alter possibilities for agency, generate new practices, and lead to different outcomes. Situational Organisation thus provides an important mediating dimension within the NEF configuration without being treated as a deterministic causal stage.

#### 3.1.5 Outcome organisation

Outcome Organisation concerns how situations, problems, conflicts, challenges, achievements, or other consequential developments are addressed and what follows from them within the narrative world. Narrative comprehension research has emphasised the importance of consequences and outcomes in understanding the coherence and development of narrative events; readers connect characters’ goals, actions, and consequences to construct an integrated representation of what happens in a story (Lynch and van den Broek, 2007; Paris and Paris, 2003). From a narrative-structural perspective, outcomes also contribute to the transformation of situations and progression of narrative events (Mandler and Johnson, 1977). NEF extends this perspective by treating the organisation of outcomes as a distinct dimension of the narrative world rather than reducing outcomes to the final event or moral lesson of a story.

The central analytical question of Outcome Organisation is: How are the situations that emerge within the narrative world addressed, transformed, resolved, continued, or otherwise carried forward, and what follows from them?

Outcome Organisation therefore examines how consequential situations are handled and what kinds of consequences, transformations, continuities, or developments follow. A situation may be resolved, partially resolved, transformed, managed, avoided, left unresolved, or carried forward into another situation. Outcomes may involve recognition of achievement, restoration or alteration of relationships, learning, loss, changes in circumstances, continuation, or new challenges. They may be immediate or gradual, individual or collective, expected or unexpected, and may include multiple or competing outcomes rather than a single definitive resolution.

Outcome Organisation should be distinguished from Situational Organisation: Situational Organisation concerns the emergence and development of problems, conflicts, incidents, challenges, and changing circumstances, whereas Outcome Organisation concerns how those situations are subsequently addressed and what follows from them. Conflict resolution is therefore one possible form of outcome organisation rather than an independent dimension. Similarly, NEF does not introduce a separate category of outcome attribution; the reasons an outcome occurs can be understood through the configuration of agency, relationships, practices, and situations that precedes and accompanies it.

Outcome Organisation should also not be equated with a predetermined moral lesson or with the assumption that an outcome represents the “correct” response to a situation. A narrative may reward, punish, complicate, reverse, question, or leave ambiguous the consequences of characters’ actions. The analytical concern is how consequences are organised within the narrative world and connected to subsequent developments. Outcome Organisation therefore provides an important bridge to Representation Organisation, through which the narrative frames, evaluates, emphasises, or carries forward what has happened.

#### 3.1.6 Representation organisation

Representation Organisation concerns how characters, relationships, practices, situations, and outcomes are selected, framed, emphasised, evaluated, connected, and carried forward within the narrative. Narratological approaches have long recognised that narratives do not merely present events but organise information through choices concerning perspective, narration, emphasis, description, and the distribution of narrative information (Herman, 2009; Bal, 2017). Such choices influence what readers are invited to notice and how events, characters, and developments become intelligible within the storyworld. NEF extends this perspective by treating representation as an organisational dimension operating across the preceding dimensions rather than as a purely stylistic feature of narration.

The central analytical question of Representation Organisation is: How are characters, relationships, practices, situations, and outcomes represented, framed, evaluated, emphasised, and carried forward within the narrative world?

Representation Organisation therefore examines what is foregrounded or backgrounded, how significance is attributed, whose perspectives or experiences receive emphasis, how consequences are presented, and how earlier events are connected to subsequent meaning. Representation may reinforce, complicate, qualify, or challenge the significance suggested by preceding narrative developments. A narrative may, for example, foreground a character’s effort rather than ability, emphasise cooperation rather than individual achievement, or present an outcome as justified, problematic, or deliberately ambiguous.

Representation Organisation should be distinguished from the other dimensions while remaining closely connected to them. Agency Organisation concerns how characters are positioned as agents; Relational Organisation concerns how agents are positioned in relation to one another; Practice Organisation concerns how their capacities and relationships are enacted; Situational Organisation concerns the situations that emerge and develop; and Outcome Organisation concerns how those situations are addressed and carried forward. Representation Organisation concerns how this developing configuration is made available to the reader as a particular representation of what has happened and what it may mean.

Representation Organisation is therefore not equivalent to a moral lesson, narrator’s explicit statement, or single thematic message. A narrative may represent significance implicitly through recurring emphasis, character response, contrast, consequence, perspective, or later reference to earlier events, and representation may remain open-ended or contradictory. Its analytical significance lies in how the narrative organises the visibility and significance of the preceding configuration, thereby completing the six-dimensional analytical space without functioning as a final or deterministic stage.

### 3.2 Narrative Ecological Configuration

The six dimensions described above are analytically distinguishable, but they do not operate as independent features of a narrative world. Narrative comprehension involves constructing relations among characters, actions, events, goals, and other elements through which readers develop coherent representations of storyworlds (Herman, 2009; Lorch and Hayden, 2020). From a broader narratological perspective, the significance of narrative elements also emerges through their relations within the world and the situations that the narrative makes available to the reader (Caracciolo, 2014). Building on this relational understanding, the Narrative Ecological Configuration (NEC) refers to the integrated pattern produced through the organisation and interaction of Agency Organisation, Relational Organisation, Practice Organisation, Situational Organisation, Outcome Organisation, and Representation Organisation within a particular narrative world.

The central analytical question of NEC is: How are the six dimensions organised and interconnected within a particular narrative world, and what configuration emerges from their interaction?

An NEC is not simply the aggregate of six dimension-level descriptions. It concerns the relational pattern formed among those dimensions within a narrative world. Such configurations may involve mutual reinforcement, tension, constraint, transformation, complementarity, or contradiction. For example, an agent’s capacities may be enabled or constrained by relationships; those relationships may shape practices; practices may generate or alter situations; situations may modify subsequent agency or relationships; and representation may reinforce, qualify, or challenge the significance of these developments. A configuration therefore becomes analytically meaningful when the dimensions are considered in terms of how they condition, modify, reinforce, constrain, or reinterpret one another, rather than merely being listed alongside one another.

An NEC should also be distinguished from more conventional ways of analysing narrative. Plot, character, theme, and moral analysis each foreground important aspects of a narrative, but none is equivalent to examining how multiple dimensions of the narrative world are organised and interact as a configuration. Table 2 summarises this distinction.

**Table 2:** Distinguishing Narrative Ecological Configuration from conventional narrative analyses.

Analytical focus	Primary question	What it foregrounds	Relationship to NEC
Plot	What happens, and in what sequence?	Events, progression, and causal development	NEC considers how the organisation of the narrative world shapes the significance and relations of events.
Character analysis	Who are the characters and how are they constructed?	Character traits, goals, motivations, and development	NEC considers character agency and relationships as dimensions within a broader configuration.
Theme	What broader ideas or meanings does the narrative convey?	Recurring ideas, values, or meanings	NEC examines the organisational conditions through which meanings may emerge or acquire significance.
Moral lesson	What lesson or value does the narrative communicate?	Explicit or inferred normative meanings	NEC does not assume a single lesson; it examines how outcomes and their representation are organised.
Narrative Ecological Configuration (NEC)	How are agency, relationships, practices, situations, outcomes, and representation organised and interconnected?	The configuration produced through their interaction within the narrative world	Provides an additional integrative analytical level rather than replacing other forms of narrative analysis.

The distinction is not intended to position NEC as a replacement for established narrative or cognitive approaches. Rather, NEC occupies a different analytical level by characterising how multiple dimensions of narrative-world organisation are configured together. Story-world and narrative-situation approaches provide accounts of represented worlds and the situations and events that occur within them; approaches concerned with transportation, engagement, and narrative persuasion primarily address readers’ involvement and responses; mental models, situation models, scripts, and schemas concern cognitive representations, learning, and generalisation; and social-cognitive, moral-learning, narrative-identity, and theory-of-mind approaches address particular psychological, social, or developmental processes. Related approaches to epistemic cognition, ideology, and worldview address orientations toward knowledge, belief, values, or social reality, while characterisation and representation approaches examine how particular agents, groups, or perspectives are constructed and made salient. NEF brings these levels into relation by first specifying the organisation of the narrative environment through NECs and then proposing, rather than assuming, that recurring configurations may become relevant to reader-level processes. Its distinctive contribution therefore lies not in any single dimension, but in providing an integrative analytical account of how agency, relationships, practices, situations, outcomes, and representation are organised and interact within a narrative world. An extended comparison of this configuration-centred perspective with established narrative and cognitive approaches is provided in Appendix B.

The following example illustrates how this additional analytical level can be applied by identifying the six dimensions within a narrative and subsequently considering them as an integrated configuration. The example is intentionally simplified and is not intended as an exhaustive interpretation of the narrative (Table 3).

**Table 3:** Illustrative mapping of the six dimensions in *The Lion and the Mouse.*

Dimension	Illustrative organisation in the narrative
Agency Organisation	The lion is characterised through physical strength and power, while the mouse, despite physical vulnerability, is positioned as capable of initiative and effective action.
Relational Organisation	The relationship begins with a pronounced power asymmetry between predator and prey but develops toward reciprocity and mutual dependence.
Practice Organisation	The lion’s decision to spare the mouse and the mouse’s later effort to free the lion show how character qualities and their relationship become enacted through decisions, actions, and treatment.
Situational Organisation	The lion’s entrapment creates a problematic situation in which the earlier power relationship is temporarily reversed and the mouse becomes consequential to the lion’s circumstances.
Outcome Organisation	The problematic situation is addressed through the mouse’s intervention, resulting in the lion’s release and a transformation of the significance of their earlier interaction.
Representation Organisation	The narrative foregrounds the reversal of dependence and gives significance to an action that initially appears insignificant, carrying this significance into the narrative’s broader meaning.

In this example, the dimensions are interconnected rather than independent. The lion’s greater physical capacity establishes an initial relational asymmetry, which shapes the significance of his decision to spare the mouse. The mouse’s later intervention demonstrates how that relationship can be transformed through practice when a changed situation alters the relevance of their respective capacities. The resulting outcome changes the significance of the earlier interaction, while the narrative’s representation foregrounds the reversal of dependence and gives it continuing significance. The example therefore illustrates that an NEC is not produced by adding six dimension-level observations, but by examining the relations, tensions, transformations, and mutual influences among the dimensions through which a narrative world develops.

As Figure 2 illustrates, the configuration of the six dimensions should not be understood as a fixed structure or linear pathway. Rather, the dimensions may be interconnected in different ways within a particular narrative world, with changes in one dimension potentially reshaping others. Their relative prominence and organisation may vary across narratives and across developments within the same narrative. NEC therefore captures an integrated pattern of relations among the dimensions rather than a predetermined arrangement of narrative features.

**Figure 2:**
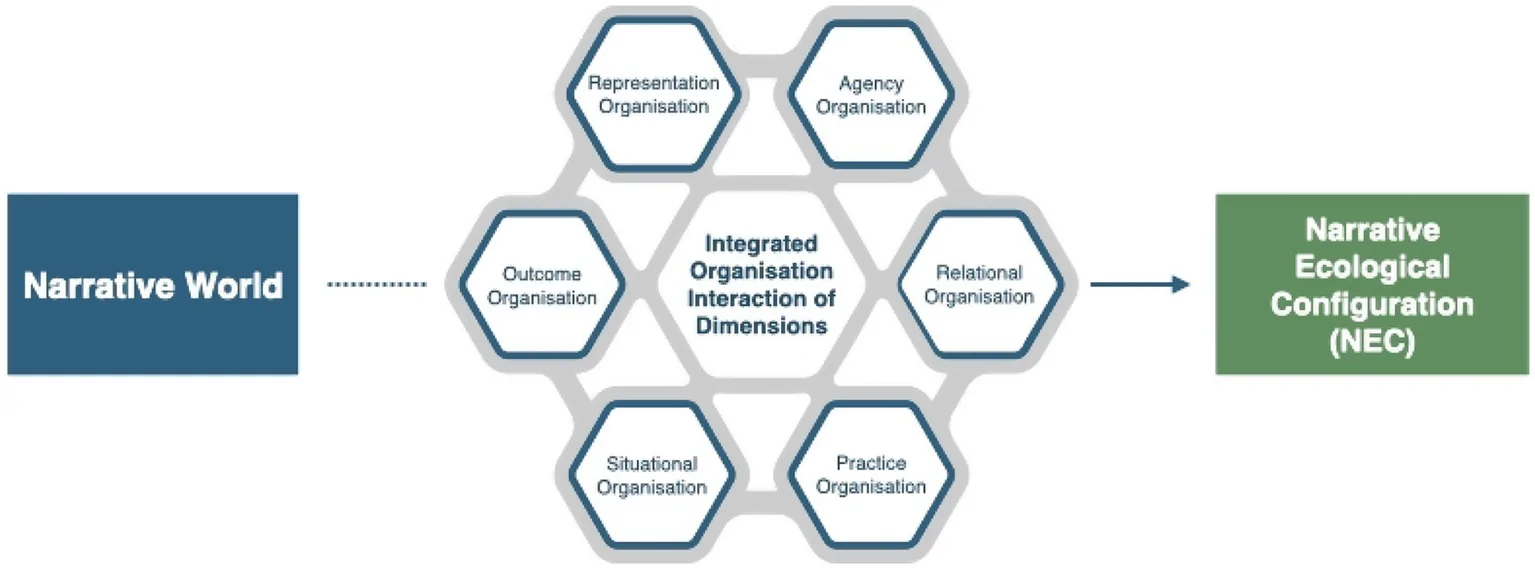
Integrated Organisation of the Six Dimensions within a Narrative Ecological Configuration. The figure represents the six dimensions as analytically distinguishable but interconnected aspects of a narrative-world configuration. The connectors indicate proposed associations rather than deterministic or causal relationships; the figure does not imply a fixed sequence through which the dimensions necessarily operate.

NECs should therefore be understood as variable and continuous configurations rather than fixed categories or typologies. Different tendencies may coexist within the same narrative, and the relative prominence or organisation of a dimension may change as the narrative develops. Agency may be enabled or constrained by relationships and situations; practices may alter existing relationships or generate new situations; outcomes may transform subsequent agency and relationships; and representation may foreground, qualify, or reinterpret the significance of earlier developments. The dimensions consequently operate through reciprocal and potentially recursive relations rather than through a fixed linear sequence.

The concept of NEC shifts the analytical focus from asking what individual narrative elements are present to asking how they are organised in relation to one another. Although individual characters, relationships, actions, situations, or outcomes may be examined separately, their significance becomes more visible when considered as an interconnected configuration. NEC therefore provides an analytical construct through which such configurations can be described and compared across narratives.

For qualitative application, the analysis proceeds from textual evidence to dimension-level characterisation and then to examination of relations among dimensions. Analysts may identify multiple tendencies within a dimension when the narrative provides evidence for their coexistence or variation across developments. Where one tendency is described as more prominent, its designation should be supported by its recurrence, narrative prominence, consequences for subsequent developments, or explanatory relevance within the analysed narrative rather than by a predetermined numerical threshold. Where more than one interpretation remains plausible, analysts should retain the alternatives when supported by the textual evidence rather than force a single categorisation. The subsequent NEC synthesis should then make explicit the relations among the identified tendencies, including patterns of reinforcement, tension, constraint, transformation, complementarity, or contradiction. These guidelines are intended to increase analytic transparency while retaining the interpretive character of qualitative analysis; systematic procedures for inter-rater agreement, coder adjudication, and formal comparison of NECs remain matters for future empirical development.

Importantly, NEF does not propose that a particular NEC produces a predetermined interpretation in a reader. Rather, recurring NECs across narrative experiences may make available recurring ways of organising agency, relationships, practices, situations, outcomes, and their representation. The possible significance of such recurrence for interpretation is addressed in the following section.

Further illustrative applications of the six dimensions, including the development of Narrative Ecological Profiles and overall Narrative Ecological Configurations across diverse narrative traditions, are provided in Appendix C. These worked examples are intended to demonstrate the descriptive and comparative application of NEF rather than to provide empirical validation or evidence of reader-level effects.

### 3.3 Recurring NECs and interpretive orientations

A single Narrative Ecological Configuration (NEC) does not determine how a reader interprets a narrative. Its potential significance lies in recurrence: when readers encounter similar configurations across multiple narratives, they may repeatedly encounter related ways of organising agency, relationships, practices, situations, outcomes, and their representation. Such recurring configurations may therefore become associated with broader interpretive orientations—relatively general tendencies through which readers approach and make sense of subsequent narrative or social situations. This proposition is offered as a conceptual extension of theoretical work on narrative meaning-making and the organisation of knowledge and expectations (Bruner, 1990, 2002). However, interpretive orientations are not treated as fixed beliefs, moral values, or direct products of particular stories. They are conceptualised as possible tendencies in interpretation associated with recurring configurations, while their emergence and use may also be influenced by readers’ prior knowledge, experiences, cultural contexts, and subsequent encounters. NEF therefore proposes an association rather than a deterministic pathway: whether and how recurring configurations are recognised, generalised, and used in interpretation remains an empirical question.

NEF distinguishes among the narrative configuration itself, readers’ cognitive representations, schemas, expectations, and broader interpretive orientations. An NEC is a property of narrative-world organisation: it describes the relational configuration formed among the six dimensions. A cognitive representation, by contrast, refers to a reader-level representation that may be constructed through narrative engagement. Within this broader category, a schema is an organised knowledge structure that can guide interpretation of subsequent instances (Anderson, 1977; Bartlett, 1932; Rumelhart, 1980), whereas an expectation concerns an anticipatory orientation toward what may occur or how a situation may develop based on prior knowledge or experience. Interpretive orientations are broader tendencies through which subsequent narrative or social situations may be approached and understood. These constructs are therefore not interchangeable: an NEC is not itself a schema, expectation, belief, or worldview orientation, and identifying an NEC does not demonstrate that a corresponding reader-level representation has been formed. NEF instead proposes a possible relationship across these levels, the psychological development and durability of which require empirical investigation.

The term ‘worldview’ has been used to describe broader systems through which people understand and interpret reality (Naugle, 2002; Sire, 2020). Psychological accounts similarly conceptualise worldviews as broad organising perspectives through which individuals interpret and make sense of reality (Koltko-Rivera, 2004). Within this framework, a worldview orientation is broader than any single attitude, belief, value, schema, ideological position, moral judgment, social-cognitive representation, or epistemic belief (Cobern, 1991; Coats, 2020). These constructs may contribute to, express, or interact with an individual’s interpretive orientation, but none is treated as equivalent to it. Whereas an attitude or belief concerns a particular evaluative or propositional stance, a value concerns what is regarded as important or desirable, and an epistemic belief concerns assumptions about knowledge and knowing, a worldview orientation concerns a more generalised tendency in how recurring situations and relationships are interpreted across contexts. It may intersect with moral reasoning, social cognition, and ideological orientations without being reducible to any one of them. The term “relatively stable” therefore refers to potential consistency across multiple situations or experiences over time, rather than a fixed, trait-like, or irreversible disposition. NEF does not specify a particular developmental stage at which worldview orientations emerge; their differentiation, complexity, stability, and susceptibility to change remain empirical questions.

The extent to which recurring NECs may become cognitively significant is expected to vary according to developmental, cognitive, experiential, narrative, and engagement-related conditions. Age and developmental level may influence the complexity of relations readers can recognise and integrate, while cognitive and language abilities, reading proficiency, and metacognitive ability may affect access to, processing of, and reflection upon narrative information (Paris and Paris, 2003; Lorch and Hayden, 2020). Prior knowledge and experience, cultural background, educational context, and familiarity with narrative conventions may shape which relations are noticed and how recurring configurations are interpreted or generalised (Bruner, 1990; Lorch and Hayden, 2020). Narrative characteristics, including structural and representational sophistication, may affect the complexity of configurations available for recognition. The frequency and distribution of narrative exposure may affect opportunities to encounter recurring configurations, while individual differences in narrative engagement may influence attention to, involvement with, and processing of those configurations (Thompson et al., 2018; Samur et al., 2021). These conditions may therefore shape the likelihood, form, and extent of pattern recognition and generalisation, rather than operate as uniform or deterministic moderators. Their relative contributions and interactions remain empirical questions, and NEF does not assume that the proposed processes operate similarly across readers, developmental levels, narrative forms, or exposure conditions.

NEF therefore does not treat story learning primarily as the acquisition of explicit themes or moral lessons. Instead, it directs attention to the organisation of narrative experience and proposes that what may become significant for readers is not only what a story says, but recurring patterns in how its narrative world is organised.

### 3.4 Pattern Extraction and Pattern Abstraction

When similar Narrative Ecological Configurations recur across narrative experiences, readers may recognise regularities that extend beyond individual stories. Pattern Extraction refers to the recognition of recurring relationships or organisational regularities across NECs, whereas Pattern Abstraction refers to the integration and generalisation of such regularities into more general cognitive representations that may be brought to subsequent experiences. This distinction is informed by research on schemas and schema induction, which suggests that knowledge structures can be formed across multiple experiences and subsequently used to organise or interpret new instances (Bartlett, 1932; Rumelhart, 1980; Ghosh and Gilboa, 2014). Research on analogical learning likewise indicates that comparison across examples can support the induction of more general relational structures that facilitate subsequent transfer (Gick and Holyoak, 1983; Chen and Mo, 2004).

Within NEF, the relevant regularities are not restricted to conventional story structures or repeated sequences of events. They may concern recurring patterns in the organisation and interaction of the six dimensions, such as relations between agency and relationships, practices and situations, or situations and outcomes. Pattern Extraction therefore concerns recognition of similarities or regularities across NECs, whereas Pattern Abstraction concerns the potential representation and generalisation of such recurring relations at a more general level across experiences. In this respect, NEF extends the idea of recurring narrative structure beyond conventional story schemas by proposing that readers may potentially become sensitive to broader configurations in how narrative worlds organise agency, relationships, practices, situations, outcomes, and representation.

The proposed processes are consistent with research showing that generalisation across experiences depends on the structural relations learners recognise across instances. Exposure to multiple examples can support broader and more flexible representations, whereas restricted experience may produce narrower representations (Chen and Mo, 2004). Research on transfer further indicates that applying previously acquired knowledge to new contexts is not automatic but depends on characteristics of the prior learning experience and the relation between prior and subsequent contexts (Barnett and Ceci, 2002). Accordingly, NEF does not assume that repeated exposure to similar NECs necessarily produces abstraction or that an abstracted representation will necessarily be transferred to subsequent experiences.

Pattern Extraction and Pattern Abstraction are therefore analytically distinguishable but theoretically interconnected. This distinction does not establish that they are psychologically independent processes. Pattern Extraction concerns the recognition of recurring structure across NECs, whereas Pattern Abstraction concerns the potential generalisation and integration of that recognised structure beyond individual narrative instances. The relationship is not proposed as a rigid or deterministic sequence: recognition may provide a basis for abstraction, while more generalised representations may subsequently influence which relations are noticed or recognised as similar in new experiences. The relationship may therefore be reciprocal, and the two processes may involve deliberate or less explicit forms of learning, comparison, generalisation, and memory. NEF does not specify whether either process is necessarily conscious or implicit. They are therefore distinguished from ordinary comprehension and inference: comprehension concerns constructing a coherent representation of a particular narrative, whereas inference concerns interpreting or filling information within that narrative; Pattern Extraction and Pattern Abstraction concern recognition and potential generalisation of recurring organisational relations across narrative instances. Whether these processes can be empirically distinguished from related forms of comprehension, inference, schema induction, abstraction, or generalisation remains an empirical question.

Importantly, Pattern Extraction and Pattern Abstraction are theoretical components of NEF rather than empirically established psychological mechanisms demonstrated by the present study. The present analysis can identify recurring NECs at the level of narrative-world organisation, but it does not establish that readers extract or abstract those configurations, retain them in memory, generalise them across experiences, or transfer them to subsequent situations. Nor does NEF assume that abstraction necessarily results in a particular schema, expectation, or interpretive orientation. These possibilities require reader-based empirical investigation.

Figure 3 should therefore be understood as a conceptual representation rather than a model of an established psychological sequence. Pattern Extraction concerns the recognition of recurring relations, tendencies, or regularities across narrative experiences, whereas Pattern Abstraction concerns the organisation of such regularities at a more general level that may extend beyond the particular narrative instances from which they were derived. The two processes are analytically distinguishable but need not be understood as strictly separate or universally sequential operations; abstraction may draw attention back to particular instances, and subsequent narrative experiences may modify or reorganise previously extracted patterns. The figure therefore represents a proposed theoretical relationship rather than a fixed psychological mechanism or empirically established developmental pathway.

**Figure 3:**
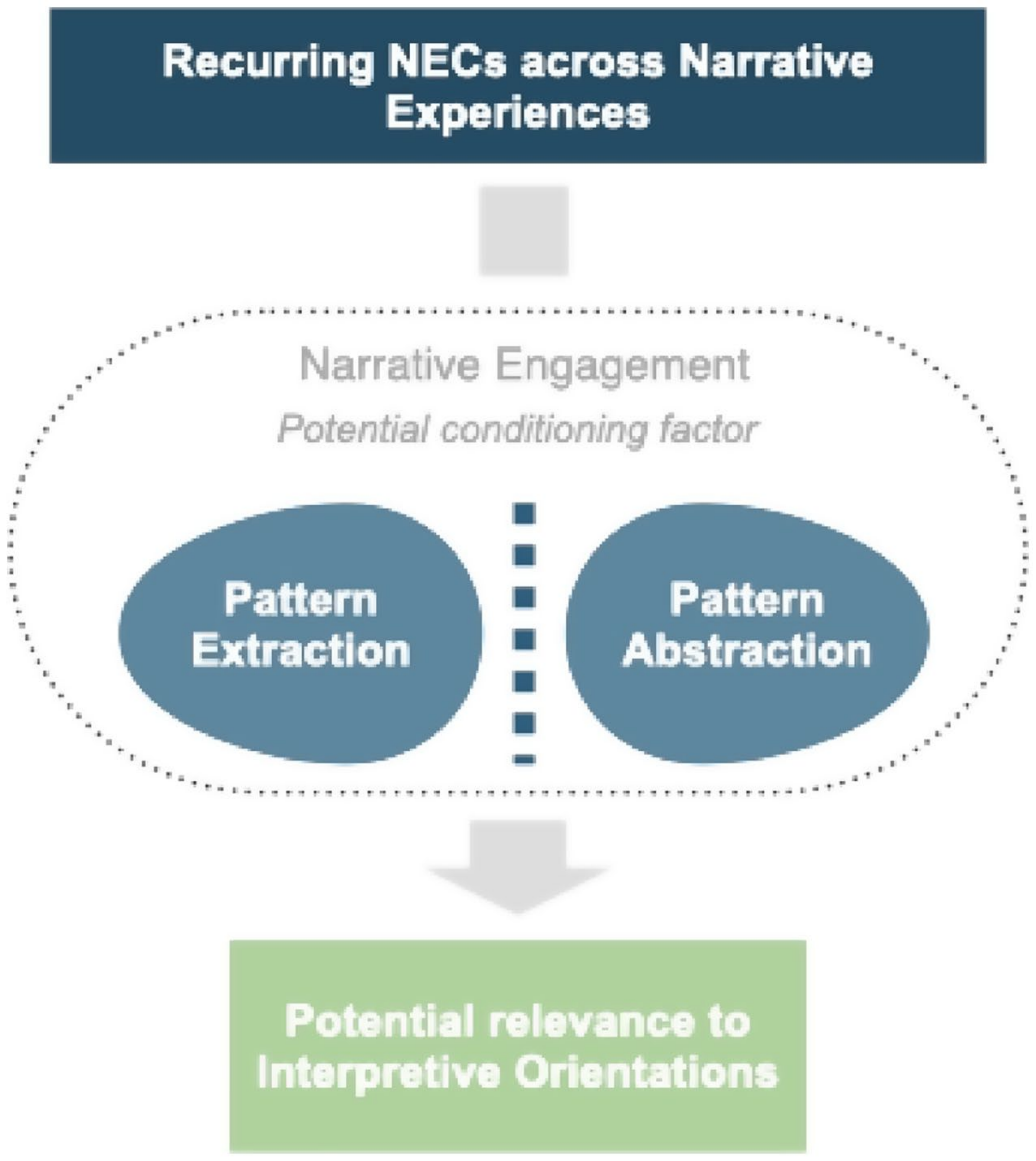
Recurring Narrative Ecological Configurations and Proposed Cognitive Processes. The figure represents Pattern Extraction and Pattern Abstraction as theoretically distinguishable but interconnected processes. Pattern Extraction concerns the recognition of recurring organisational regularities across NECs, whereas Pattern Abstraction concerns their potential integration and generalisation into more general cognitive representations. The connectors indicate proposed relationships rather than established causal or deterministic pathways. The figure does not imply that repeated exposure necessarily produces abstraction, schema development, or particular interpretive outcomes.

At this stage, NEF does not claim that exposure to a particular NEC necessarily results in Pattern Extraction or Pattern Abstraction, nor that these processes inevitably produce particular schemas, expectations, or worldview orientations. Narrative engagement may represent one condition influencing these processes, but its contribution should not be treated as unitary. Engagement may include sustained attention, emotional involvement, transportation, identification, perspective taking, and reflective engagement (Thompson et al., 2018; Samur et al., 2021), and these dimensions need not have equivalent relationships with Pattern Extraction and Pattern Abstraction. Sustained attention, for example, may increase opportunities to notice recurring relations, whereas emotional involvement, identification, or transportation may influence their salience, and reflective engagement may support deliberate comparison or evaluation across experiences. Within NEF, engagement may therefore potentially condition both processes, although the relative contribution of different forms of engagement remains an empirical question. Future research could examine these dimensions through appropriate behavioural, task-based, observational, or validated self-report measures, depending on the aspect of engagement under investigation. Importantly, NEF does not assume that greater engagement necessarily produces beneficial or desirable outcomes. Where recurring narrative configurations contain stereotypical, exclusionary, or otherwise problematic patterns, stronger engagement could potentially increase attention to, elaboration of, or receptivity to such patterns. Narrative engagement is therefore treated as a potential conditioning factor rather than as a uniformly facilitative mechanism, a separate stage in the framework, or an empirically established moderator.

### 3.5 Configurational understanding and future empirical inquiry

Pattern Extraction and Pattern Abstraction describe how recurring narrative regularities may become cognitively available, but they should not be equated with readers’ conscious identification of the six dimensions. Configurational Understanding refers more specifically to the reader’s capacity to recognise and integrate how multiple dimensions of a narrative world interact to produce a particular configuration. It therefore concerns understanding relations among dimensions, rather than recognition of isolated narrative features.

This distinction provides a basis for future empirical research. NEF proposes that configurational understanding may be associated with deeper narrative comprehension, particularly where readers must integrate characters, relationships, practices, situations, outcomes, and their representational significance. This proposition remains to be tested rather than assumed. Future studies can therefore examine whether stronger configurational understanding is associated with more successful comprehension of narrative situations, causal relations, character motivations, and broader narrative meaning.

The proposed construct also provides a potential bridge between the conceptual and empirical phases of the research programme. The present paper establishes NEC as a narrative-level configuration and proposes Pattern Extraction and Pattern Abstraction as possible processes through which recurring configurations may become available for interpretation. Subsequent empirical research can operationalise Configurational Understanding and examine its relationship with narrative comprehension. In this way, NEF moves from a conceptual account of narrative-world organisation toward testable questions about how readers understand and respond to recurring narrative configurations.

## 4 Implications and future research

Having established the Narrative Ecological Framework as a conceptual framework for examining the organisation of narrative worlds, this section considers its broader theoretical and educational significance and identifies directions for empirical development. NEF shifts attention from isolated narrative elements or explicit story messages toward configurations produced through interactions among agency, relationships, practices, situations, outcomes, and representation. Its significance therefore lies not in claiming that narratives produce predetermined cognitive or interpretive effects, but in providing a conceptual basis for examining how recurring narrative configurations may become relevant to readers’ interpretive processes. The section first considers the framework’s theoretical contribution, then its potential educational implications, and finally the empirical research needed to examine its proposed relationships.

### 4.1 Theoretical implications

The Narrative Ecological Framework extends existing approaches to narrative learning by shifting analytical attention from individual narrative elements, themes, or explicit lessons toward the organisation and interaction of multiple dimensions within narrative worlds (Herman, 2009; Lorch and Hayden, 2020). Its principal conceptual contribution is the Narrative Ecological Configuration (NEC), which provides an integrative analytical level for examining how agency, relationships, practices, situations, outcomes, and representation are organised together. This configurational perspective enables comparison of narratives that may share similar themes or plots but organise their narrative worlds in substantially different ways.

A second contribution is the distinction between individual NECs and recurring NECs. NEF does not propose that a single narrative determines a reader’s interpretation. Rather, repeated encounters with similar configurations may make particular organisational regularities increasingly available for recognition and interpretation. The framework consequently provides a conceptual bridge between narrative-world organisation and the possibility of broader interpretive orientations, while leaving the strength and nature of this relationship open to empirical investigation.

A third contribution concerns the proposed distinction between Pattern Extraction and Pattern Abstraction. These processes provide a possible cognitive account of how recurring configurations may become represented at a more general level across narrative experiences (Gick and Holyoak, 1983; Chen and Mo, 2004). Importantly, NEF treats them as proposed cognitive processes rather than established causal mechanisms. This distinction allows the framework to connect recurring narrative organisation with reader cognition without assuming that repeated narrative exposure automatically produces particular cognitive or interpretive outcomes.

Finally, the framework introduces Configurational Understanding as a potential reader-level construct for future investigation. Whereas NEC describes the organisation of the narrative world, Configurational Understanding concerns the reader’s capacity to recognise and integrate interactions among its dimensions. This distinction separates the narrative-level configuration from the reader-level capacity through which it may be understood, providing a basis for empirical research examining whether stronger configurational understanding is associated with deeper narrative comprehension.

Taken together, these constructs define NEF as a conceptual and testable framework rather than a validated causal model. Table 4 distinguishes the analytical constructs that the present framework introduces from the psychological processes and reader-level outcomes whose operation, measurement, and relationships remain to be established empirically.

**Table 4:** Integrative summary of core NEF constructs and proposed empirical status.

Construct	Analytical level	Concise definition	Proposed role	Empirical status/possible investigation
Six Narrative-World Dimensions	Narrative-world	Agency, relationships, practices, situations, outcomes, and representation as analytically distinguishable aspects of narrative-world organisation	Provide the dimensions through which narrative worlds are analysed	Provisional analytical architecture; coding reliability and cross-context applicability require testing
Narrative Ecological Configuration (NEC)	Narrative-world	Integrated pattern formed through the organisation and interaction of the six dimensions within a narrative world	Provides the configuration-centred unit for describing and comparing narratives	Newly proposed analytical construct; reliability and construct validity require empirical investigation
Recurring NECs	Across narratives	Similar or recurring configurations encountered across multiple narrative experiences	Provide potential recurring organisational regularities for reader-level processing	Empirical identification and recurrence require investigation
Pattern Extraction	Reader/cognitive process	Recognition of recurring organisational regularities across NECs	Potentially makes recurring relations available for further cognitive organisation	Theoretical construct informed by schema induction, abstraction, and related learning research; psychological status and measurement remain open
Pattern Abstraction	Reader/cognitive process	Potential integration and generalisation of recognised regularities into more general representations	Potentially extends recurring patterns beyond individual narrative instances	Theoretical construct; independence from Pattern Extraction and measurement require empirical investigation
Configurational Understanding	Reader-level capacity	Capacity to recognise and integrate interactions among the six dimensions within a narrative world	Potentially supports deeper integration of narrative relations and comprehension	Newly proposed reader-level construct; operationalisation and validity require testing
Interpretive Orientation	Reader-level	Relatively general tendency through which subsequent narrative or social situations may be approached and understood	Potential outcome/contextual consequence associated with recurring configurations	Proposed association only; stability, change, measurement, and causal contribution require investigation
Worldview Orientation	Broader reader-level orientation	A relatively generalised interpretive tendency concerning recurring situations and relationships across contexts	Represents a broader possible orientation that may intersect with beliefs, values, schemas, and other constructs	No validated measure proposed; developmental stability, change, cultural variation, and narrative contribution remain empirical questions

### 4.2 Educational and pedagogical implications

The Narrative Ecological Framework offers a broader basis for the analysis and selection of narratives in educational contexts. Rather than focusing exclusively on plot, theme, moral lessons, or isolated literary features, educators can use the six dimensions to examine how a narrative organises agency, relationships, practices, situations, outcomes, and representation, and how these dimensions interact within an NEC. Such analysis may support more systematic comparison of narratives and help educators consider the configurations that different texts make available, rather than selecting narratives solely on the basis of theme or explicit moral content. It may also encourage learners to justify interpretations by attending to relationships among narrative elements rather than relying only on explicit messages or personal impressions. This extends established approaches to literary and narrative learning that emphasise readers’ active construction of meaning (Rosenblatt, 1978; Nikolajeva, 2014).

The framework may also support classroom discussion that moves from what happened to how the narrative organised what happened. Questions about who is positioned as capable of acting, how relationships shape practices, how practices generate situations, how outcomes are handled, and how developments are represented can encourage integrative and evidence-based interpretation. Such discussion may provide opportunities to develop configurational understanding by directing learners’ attention to relationships among dimensions rather than to isolated narrative features. However, NEF should not yet be presented as a validated instructional intervention or assessment tool. Its pedagogical value remains a proposition for future investigation, particularly through studies examining whether instruction that develops configurational understanding is associated with deeper narrative comprehension.

### 4.3 Future empirical research

The Narrative Ecological Framework generates several directions for empirical investigation. Its evidential status should be distinguished across four levels. First, some components draw on established findings and theoretical accounts concerning narrative comprehension, schema formation, pattern learning, generalisation, memory, transfer, and interpretation (Mandler and Johnson, 1977; Lorch and Hayden, 2020; Gick and Holyoak, 1983; Barnett and Ceci, 2002). Second, NEF extends these perspectives by proposing that recurring configurations in narrative-world organisation may provide structured experiences from which relational regularities could be recognised and generalised. Third, NEF newly proposes Narrative Ecological Configuration, Pattern Extraction, Pattern Abstraction, and Configurational Understanding as components of an integrated explanatory architecture. Fourth, the proposed relationships among recurring NECs, these reader-level processes, schemas, expectations, and interpretive orientations remain empirical questions rather than established effects. Future studies can therefore test specific components independently—for example, NEC coding reliability, recurring configurations across narratives, associations between repeated exposure and pattern recognition or abstraction, and relationships among these processes and reader-level measures—without presuming that the complete pathway operates uniformly or causally.

First, future research can examine whether the six dimensions can be identified consistently across narratives and whether their interaction can be represented as reliable Narrative Ecological Configurations (NECs). Such work could develop coding protocols, test inter-rater agreement, and compare NECs across genres, narrative forms, and cultural contexts, with subsequent studies examining their relevance across age groups. This would establish the analytical reliability of the framework before examining its reader-related implications.

Second, research can investigate the proposed reader-side construct of Configurational Understanding. Studies can examine whether learners recognise not only individual narrative features but also interactions among agency, relationships, practices, situations, outcomes, and representation, and whether stronger configurational understanding is associated with deeper narrative comprehension, including integration of character motivations, causal relations, situational developments, and outcomes (Lynch and van den Broek, 2007; Lorch and Hayden, 2020; Paris and Paris, 2003). Future developmental research should examine age or developmental stage alongside cognitive and language ability, reading proficiency, metacognitive ability, prior knowledge, cultural background, narrative sophistication, frequency of narrative exposure, and individual differences in narrative engagement. Such research could examine age or developmental stage alongside these conditions to test whether they are associated with differences in the recognition, abstraction, or interpretation of recurring NECs and whether their contributions vary across developmental periods or narrative conditions.

Research on worldview orientations would require reader-level operationalisation rather than inference from narrative texts alone. Future studies could examine candidate orientations through repeated measures of interpretive expectations across multiple situations or narrative experiences, particularly in relation to agency, authority and relationships, knowledge, conflict, outcomes, and belonging. Evidence of change would require systematic differences across repeated assessments that extend beyond variation in responses to individual narratives and are sustained across comparable situations or contexts. Studies should also examine consistency over time and consider relevant background and contextual influences, including prior knowledge and experience, family and peer environments, educational experiences, cultural context, and other forms of socialisation. Longitudinal, comparative, or experimental designs, where appropriate, could help distinguish associations with narrative exposure from broader developmental and contextual influences. NEF therefore does not presently provide a validated measure of worldview orientation; its operationalisation, stability, change, and relationship to narrative experience remain open to empirical investigation.

Finally, longitudinal and comparative research can examine whether repeated exposure to particular NECs is associated with recurring interpretive orientations and whether measures or indicators related to Pattern Extraction and Pattern Abstraction help account for such associations. Such research should consider readers’ prior knowledge, cultural and educational experiences, and differences in narrative engagement rather than attributing observed patterns to narrative exposure alone. Thus, the present framework generates testable propositions rather than establishing causal developmental effects.

## 5 Limitations

The Narrative Ecological Framework is presently a conceptual framework rather than an empirically validated model. The six dimensions and the construct of Narrative Ecological Configuration provide an analytical vocabulary for examining narrative-world organisation, but their reliability and applicability across narratives, genres, age groups, and cultural contexts remain to be established. Future research will therefore need to develop and test systematic coding procedures and examine whether the dimensions can be identified consistently across analysts and narrative materials. The analytical materials provided in Appendices A–C illustrate the proposed coding procedure, conceptual positioning, and application of NEF across narrative cases, but do not constitute empirical validation of the framework or a validated assessment instrument.

A further limitation concerns the possibility that some formulations within the six dimensions reflect assumptions derived from particular intellectual or cultural traditions. Concepts such as agency, authority, knowledge, inclusion, and successful outcomes may be organised and valued differently across cultural contexts. The framework therefore should not be assumed to be culturally neutral, and its applicability across diverse narrative traditions requires critical examination of whether the dimensions are interpreted equivalently or require culturally situated modification.

A second limitation concerns the proposed relationship between recurring NECs and interpretive orientations. NEF does not establish that particular narrative configurations produce particular interpretive tendencies. Readers’ interpretations are shaped by multiple influences, including prior knowledge, experience, educational and cultural contexts, and individual differences. The interpretation of recurring configurations may therefore vary across readers and contexts because narrative experience is embedded within broader systems of knowledge, experience, and socialisation. Narrative engagement may likewise influence the extent to which recurring configurations are noticed and organised, although its role remains a theoretical proposition rather than an established moderating effect. NEF therefore proposes a possible association requiring empirical investigation rather than a deterministic developmental pathway.

Finally, Pattern Extraction, Pattern Abstraction, and Configurational Understanding remain theoretically specified rather than empirically established constructs. Their relationships to one another, particularly the proposed association between Configurational Understanding and deeper narrative comprehension, require operationalisation and testing. The present paper consequently establishes a conceptual foundation and a set of testable propositions rather than claiming to demonstrate how narrative configurations affect readers’ cognition or interpretation.

## 6 Conclusion

This paper introduced the Narrative Ecological Framework (NEF) as a conceptual approach for understanding how narrative worlds may function as organised environments in which interpretation takes place. Rather than treating stories primarily as collections of characters, events, themes, or explicit lessons, NEF focuses on how six dimensions—Agency Organisation, Relational Organisation, Practice Organisation, Situational Organisation, Outcome Organisation, and Representation Organisation—interact within narrative worlds. Their interaction produces a Narrative Ecological Configuration (NEC), providing a basis for examining narratives as integrated configurations rather than collections of isolated features.

The framework further proposes that the significance of NECs may extend beyond individual narratives when readers encounter recurring configurations across narrative experiences. Through the proposed processes of Pattern Extraction and Pattern Abstraction, such recurring configurations may become available as potential interpretive resources and may be associated with broader interpretive orientations. NEF does not, however, claim that particular narratives or configurations determine how readers interpret the world; these relationships remain open to empirical investigation.

A further contribution is the distinction between the narrative-level construct of NEC and the reader-level construct of Configurational Understanding. This distinction provides a basis for future research examining whether the ability to recognise and integrate interactions among narrative dimensions is associated with deeper narrative comprehension. The present paper therefore establishes not a completed causal model, but a conceptual architecture through which narrative organisation, recurring configurations, interpretation, and comprehension can be investigated systematically.

Overall, NEF shifts attention from asking only what a story contains or teaches toward examining how its narrative world is organised and what recurring configurations readers may encounter through narrative engagement. Its value will ultimately depends on empirical work testing the analytical dimensions and NECs, examining their consistency across narratives, operationalising Configurational Understanding, and investigating how recurring configurations relate to readers’ comprehension and interpretation.

## Data Availability

The original contributions presented in the study are included in the article/Supplementary material, further inquiries can be directed to the corresponding author/s.

## Supplementary material

Supplementary Data Sheet 1: Appendix A Illustrative Narrative Ecological Coding Manual.

Supplementary Data Sheet 2: Appendix B Conceptual Positioning of the Narrative Ecological Framework.

Supplementary Data Sheet 3: Appendix C Illustrative Applications of the Narrative Ecological Framework Across Narrative Traditions.
