# Supplementary material for: Narrative worlds as developmental environments: a Narrative Ecological Framework for understanding story learning and interpretation
Source: Front Psychol. 2026 Oct 7;17:1934346. doi: 10.3389/fpsyg.2026.1934346 (PMC13685022; doi:10.3389/fpsyg.2026.1934346)
Supplement: Supplementary file 1 [file Data_Sheet_1.PDF]

Appendix A. Illustrative Narrative Ecological Coding Manual

The Narrative Ecological Coding Manual provides an illustrative procedure for applying the Narrative Ecological Framework (NEF) to qualitative narrative analysis. Its purpose is to support systematic, transparent, and theoretically consistent interpretation of narrative worlds through the six dimensions of **Agency Organisation, Relational Organisation, Practice Organisation, Situational Organisation, Outcome Organisation, and Representation Organisation**. The manual is intended to demonstrate one possible analytical procedure and should not be interpreted as a validated coding protocol or standardised assessment instrument. Rather, it provides qualitative guidance for identifying dimension-level tendencies, examining relations among dimensions, and characterising Narrative Ecological Configurations (NECs) as integrated and variable configurations.

Table A1

Illustrative Narrative Ecological Coding Manual

Dimension	Guiding analytical question	Illustrative coding considerations	Illustrative descriptor options
**Agency Organisation**	How are characters positioned as agents capable of acting, influencing, or responding?	Identify who initiates action, influences events, shapes outcomes, or responds to changing circumstances. Consider capacities, motivations, choices, opportunities, constraints, and the distribution of agency among characters or other relevant entities.	Individually centred; Shared; Collective; Distributed; Institution-led; Constrained; Adaptive; Responsive
**Relational Organisation**	How are characters and other relevant story-world entities positioned in relation to one another?	Examine how relationships are established, maintained, negotiated, challenged, or transformed. Consider cooperation, dependence, care, trust, support, authority, opposition, competition, conflict, negotiation, affiliation, and exclusion.	Cooperative; Reciprocal; Dependent; Hierarchical; Negotiated; Distributed; Contested; Supportive; Competitive; Conflictual; Inclusive; Exclusionary
**Practice Organisation**	How are characters' capacities, relationships, knowledge, decisions, and intentions enacted through purposeful activity?	Examine actions and practices through which characters use knowledge, make decisions, solve problems, plan, cooperate, negotiate, care, assist, resist, or treat others. Consider how practices	Inquiry-oriented; Observation-based; Experience-based; Tradition-oriented; Authority-based; Deliberative; Cooperative; Negotiated; Adaptive;

Dimension	Guiding analytical question	Illustrative coding considerations	Illustrative descriptor options
		enact or modify agency and relationships.	Strategic; Problem-solving; Caring; Resistant
**Situational Organisation**	What situations emerge, develop, change, or become consequential?	Identify problems, conflicts, incidents, challenges, discoveries, opportunities, disruptions, changing circumstances, and other developments. Consider how situations are generated, intensified, transformed, or made consequential through interactions among characters and practices.	Stable; Problematic; Conflictual; Uncertain; Constraining; Enabling; Transformative; Disruptive; Opportunity-generating; Reversing
**Outcome Organisation**	How are situations addressed, transformed, resolved, continued, or otherwise carried forward, and what follows from them?	Examine how situations are resolved, transformed, managed, continued, or left unresolved. Consider consequences, recognition, loss, change, continuation, competing outcomes, and how outcomes create conditions for subsequent developments.	Resolved; Transformed; Managed; Continued; Unresolved; Reciprocal; Consequential; Loss-producing; Change-producing; Reorienting
**Representation Organisation**	How are characters, relationships, practices, situations, and outcomes represented, framed, evaluated, emphasised, and carried forward?	Examine foregrounding and backgrounding, attribution of significance, perspective, framing, emphasis, evaluation, connection, visibility, and how representation reinforces, complicates, qualifies, challenges, or carries forward the significance of developments.	Foregrounded; Backgrounded; Evaluative; Perspective-specific; Inclusive; Selective; Emphasising; Qualifying; Complicating; Challenging; Connecting

Note. The descriptor options presented above are illustrative rather than exhaustive and are intended to provide a common analytical vocabulary rather than a fixed classification system. Multiple descriptors may apply within the same dimension when supported by distinct, recurring, or otherwise consequential narrative evidence. Analysts may introduce additional descriptive formulations when the illustrative descriptors do not adequately capture a recurring pattern; however, such formulations should remain descriptive, be explicitly defined, and be anchored to identifiable narrative evidence. A descriptor should not be treated as a substantive category independent of the evidence from which it is derived. Dominant tendencies should be determined qualitatively from the **consistency, prominence, recurrence, and consequentiality** of a pattern within the narrative rather than from descriptor frequency alone. Where substantially competing tendencies are supported, they should be retained and their relation documented rather than resolved through forced categorisation. The resulting dimension-level characterisations are subsequently examined in relation to one

another to develop an integrated Narrative Ecological Profile and characterise the overall Narrative Ecological Configuration (NEC).

Illustrative Coding Procedure and Analytical Considerations

The coding procedure is intended to provide a transparent sequence for moving from narrative evidence to dimension-level characterisation and, subsequently, to an integrated NEC. The stages are analytically ordered for clarity but should not be interpreted as implying that narrative dimensions themselves operate sequentially.

Table A2

Illustrative Coding Procedure and Analytical Considerations

Step	Coding procedure	Analytical considerations
Step 1	Read the narrative as a complete Narrative World.	Consider the narrative as an organised world rather than beginning with isolated events, themes, characters, or moral lessons. Attend to recurring patterns and developments across the narrative.
Step 2	Examine each of the six dimensions using its guiding analytical question.	Treat the six dimensions as analytically distinguishable lenses. Do not assume that all six will be equally prominent or equally salient in every narrative.
Step 3	Record narrative evidence relevant to each dimension.	Ground interpretations in observable narrative features, including actions, dialogue, interactions, events, situations, outcomes, representational choices, and recurring or developing organisational patterns.
Step 4	Characterise each dimension using one or more illustrative descriptors.	Descriptors are analytic labels rather than fixed categories. More than one descriptor may apply when supported by distinct or recurring evidence. Where an existing descriptor is inadequate, an additional descriptive formulation may be introduced, provided that it is defined and anchored to identifiable narrative evidence.
Step 5	Determine dominant or coexisting tendencies within each dimension.	Judge dominance qualitatively by considering the consistency, prominence, recurrence, and consequentiality of the identified pattern. Frequency alone should not determine dominance. Where two or more tendencies are substantially supported, retain them and describe their relationship rather than forcing a single category.
Step 6	Examine relations among the six dimensions.	Identify how dimensions condition, enable, constrain, reinforce, modify, transform, complement, or come into tension or contradiction with one another. Record the narrative evidence supporting these relations.

Step	Coding procedure	Analytical considerations
Step 7	Develop the Narrative Ecological Profile.	Integrate the dimension-level tendencies and their documented relations into a coherent description of the organisation of the narrative world. The profile should distinguish dimension-level observations from the relations among them.
Step 8	Characterise the overall Narrative Ecological Configuration (NEC).	Synthesise the Narrative Ecological Profile into a concise description of the configuration produced through interaction among the six dimensions. The NEC should describe the relational pattern rather than merely reproduce six separate dimension-level descriptions.
Step 9	Compare NECs where comparative analysis is undertaken.	Compare narratives first at the dimension level and then at the level of cross-dimensional relations. Shared descriptors alone should not be treated as evidence of equivalent NECs; differences in prominence, organisation, interaction, tension, or transformation should also be considered.

Handling dominant and coexisting tendencies

The identification of a dominant tendency is intended to provide analytical focus without reducing narrative organisation to a single categorical label. A tendency may be considered dominant when it is repeatedly or consistently evident, is relatively prominent in the organisation of the narrative world, and/or has consequential effects on subsequent developments. These considerations are qualitative and should be evaluated in relation to the narrative as a whole.

A dimension may therefore contain more than one significant tendency. For example, agency may be both individually centred and constrained, or a relationship may be simultaneously hierarchical and progressively negotiated**.** In such cases, analysts should preserve the relevant combination rather than selecting one descriptor merely for the sake of categorical simplicity. The purpose of the coding is to represent the organisation of the narrative world with sufficient analytical precision, not to assign each narrative to a predetermined type.

Unit of analytical evidence. NEF does not prescribe a single universal textual unit for coding. Relevant evidence may occur at the level of an action, interaction, episode, scene, event, character relationship, or related narrative sequence, depending on the dimension and the narrative structure. Analysts should identify the smallest narrative segment sufficient to support the interpretation and should document the evidential basis for each coding decision. Dimension-level characterisations and the subsequent NEC are developed across the narrative as a whole rather than assigned mechanically to individual sentences or scenes.

Handling additional or emergent descriptors

The illustrative descriptors in Table A1 are intended to support consistency but are not intended to exhaust the possible forms of narrative organisation. Qualitative analysis may identify patterns that require more specific descriptive formulations. Terms such as *strategically adaptive agency*, *relational authority*, or *transforming authority*, for example, may be useful when they capture a recurring pattern not adequately represented by the initial descriptor vocabulary.

Such formulations should, however, satisfy three conditions:

1. They should be descriptive rather than treated as established theoretical categories.
2. Their meaning should be made explicit in the analysis.
3. Their use should be anchored to identifiable narrative evidence and, where appropriate, recurring or consequential patterns.

This allows the framework to remain sensitive to narrative variation while limiting unconstrained proliferation of interpretive labels.

Handling competing interpretations

Because NEF is intended as a qualitative analytical framework, more than one interpretation may sometimes be supported by the same narrative evidence. Analysts should therefore distinguish between evidence that is strongly recurrent, prominent, or consequential and evidence that is isolated or ambiguous. Where competing interpretations remain plausible, they should be documented rather than eliminated solely to achieve categorical consistency.

In future multi-analyst applications, such cases could be independently coded and subsequently examined through explicit comparison of the evidence, descriptor selection, and coding rationale. The present manual does not prescribe that all analysts must produce identical wording. Rather, it provides a common analytical structure through which similarities and differences in interpretation can be made explicit and examined.

Examining cross-dimensional interaction

The development of an NEC requires more than placing six dimension-level descriptions alongside one another. After the dimensions have been examined individually, analysts should explicitly consider how patterns within one dimension relate to patterns within others. Relevant relationships may include:

- **reinforcement**, where patterns in two or more dimensions strengthen one another;
- **constraint**, where one dimension limits the possibilities available within another;
- **enablement**, where one dimension creates or expands possibilities within another;
- **transformation**, where developments in one dimension alter the organisation of another;
- **complementarity**, where different dimensions operate together without being reducible to one another;
- **tension**, where dimensions support partially competing tendencies; and
- **contradiction**, where the organisation of one dimension substantially conflicts with another.

The analyst should identify the narrative evidence supporting the proposed relation rather than inferring interaction solely from the coexistence of two descriptors. For example, an initial distribution of agency may shape a relationship of authority; that relationship may organise subsequent practices; those practices may alter a situation; the altered situation may redistribute agency; and the outcome may subsequently change the meaning or representation of the original relationship. Such relations constitute part of the configuration being analysed.

Developing the Narrative Ecological Profile

The Narrative Ecological Profile is an intermediate analytical representation between dimension-level coding and the overall NEC. It should bring together:

1. the principal tendency or tendencies identified within each dimension;
2. the evidence supporting those tendencies; and
3. the principal relations identified among the dimensions.

The profile therefore provides a transparent basis for the subsequent NEC description. It should not simply repeat the descriptors from Table A1. Instead, it should show how those tendencies operate together within the narrative world.

Characterising the Narrative Ecological Configuration

The NEC should be formulated as a concise account of the integrated pattern identified through the preceding analysis. It should emphasise how the dimensions are organised in relation to one another**,** including important reinforcement, constraint, tension, transformation, complementarity, or contradiction.

The NEC should remain descriptive of the narrative world. It should not automatically be converted into a moral lesson, thematic interpretation, psychological effect, or developmental claim. Any proposed significance for readers constitutes a separate level of analysis and requires appropriate theoretical or empirical support.

Comparing Narrative Ecological Configurations

NEC comparison is initially intended as a qualitative comparative procedure rather than a numerical similarity measure. Two narratives may be considered similar when they exhibit comparable organisations across several dimensions and comparable relations among those dimensions, even when their plots, characters, settings, or surface content differ.

Conversely, narratives sharing individual descriptors may constitute different NECs when their dimensions are organised differently or when their interactions produce contrasting patterns. Comparative analysis should therefore consider both dimension-level tendencies and cross-dimensional relations**,** including reinforcement, constraint, enablement, tension, transformation, complementarity, and contradiction.

Formal procedures for representing NECs comparatively, including possible coding matrices, similarity criteria, or quantitative indices, remain matters for future methodological and empirical research.

Methodological Note

The coding procedure presented here is illustrative and intended to demonstrate the application of the Narrative Ecological Framework within qualitative narrative analysis. It is not proposed as a

validated coding protocol or standardised assessment instrument**.** The descriptors are intended to provide analytical guidance rather than fixed categories, and the resulting NECs should be understood as qualitative characterisations of narrative-world organisation.

The procedure is designed to increase transparency by making explicit the movement from narrative evidence to dimension-level tendencies, from dimension-level tendencies to cross-dimensional relations, and from those relations to an integrated NEC. Future empirical research can further evaluate the procedure through independent coding, examination of inter-rater agreement, refinement of descriptor definitions, comparison across narrative traditions and cultural contexts, and investigation of the extent to which different analysts identify comparable configurations from the same narrative material.

Narrative Text
↓
Read as a Narrative World
↓
Apply the Six Dimensions
↓
Record Narrative Evidence
↓
Characterise Each Dimension
↓
Determine Dominant / Coexisting Tendencies
↓
Examine Cross-Dimensional Relations
↓
Develop a Narrative Ecological Profile
↓
Characterise the Overall Narrative Ecological Configuration (NEC)
↓
Compare NECs, Where Applicable

Figure A1. Illustrative Workflow for Applying the Narrative Ecological Coding Manual

Note. Figure A1 illustrates the qualitative analytical workflow proposed by the Narrative Ecological Framework. The procedure begins by treating the narrative as an integrated narrative world, proceeds through systematic consideration of the six dimensions and their supporting evidence, examines relations among the dimensions, and culminates in the development of a Narrative Ecological Profile and characterisation of the overall Narrative Ecological Configuration (NEC). Comparison of NECs is shown as an optional subsequent analytical activity. The workflow is intended to support transparency and methodological discipline and should not be interpreted as a validated or standardised coding protocol.
