# Supplementary material for: Narrative worlds as developmental environments: a Narrative Ecological Framework for understanding story learning and interpretation
Source: Front Psychol. 2026 Oct 7;17:1934346. doi: 10.3389/fpsyg.2026.1934346 (PMC13685022; doi:10.3389/fpsyg.2026.1934346)
Supplement: Supplementary file 2 [file Data_Sheet_2.PDF]

Appendix B. Conceptual Positioning of the Narrative Ecological Framework

The purpose of this appendix is to clarify the distinctive analytical contribution of the Narrative Ecological Framework (NEF) in relation to established approaches to narrative analysis. NEF is not intended to replace approaches that examine plot, character, theme, moral meaning, ideology, narratological structure, or reader response. Rather, it introduces an additional analytical perspective concerned with the integrated organisation of the narrative world**.**

Many established approaches foreground particular aspects of narrative. Plot analysis examines the organisation and progression of events; character analysis examines characters, their attributes, motivations, relationships, and development; thematic and ideological approaches examine broader meanings, values, assumptions, or systems of representation; moral analysis examines ethical positions or lessons; and reader-oriented approaches examine how readers construct meaning from narrative texts. These approaches differ in their analytical questions and objects of attention and therefore provide different kinds of insight into narrative.

NEF adopts a configuration-centred perspective. Its primary analytical concern is not any single narrative element but the way multiple dimensions of narrative-world organisation are related within a particular narrative. The principal analytical unit is therefore the Narrative Ecological Configuration (NEC)**.** An NEC is derived through the systematic consideration of Agency Organisation, Relational Organisation, Practice Organisation, Situational Organisation, Outcome Organisation, and Representation Organisation and through analysis of the relations among these dimensions.

The distinction between element-centred and configuration-centred analysis is therefore analytical rather than evaluative**.** NEF does not claim that conventional approaches are inadequate or that configuration-centred analysis supersedes them. Instead, it asks a different question: How are multiple dimensions of the narrative world organised and interconnected, and what configuration emerges from their interaction?

Table B1

Conceptual Positioning of NEF in Relation to Established Narrative Analyses

Analytical approach	Primary analytical focus	Typical analytical question	Principal analytical outcome	Relationship to NEF
Plot analysis	Events, progression, and narrative sequence	What happens, and how are events organised?	Plot or structural analysis	NEF considers how the organisation of the narrative world relates to and gives significance to events.
Character analysis	Character construction, traits, goals, motivations,	Who are the characters and how are they constructed?	Character profile and analysis	NEF considers character agency and relational positioning as dimensions

Analytical approach	Primary analytical focus	Typical analytical question	Principal analytical outcome	Relationship to NEF
	relationships, and development			within a broader configuration.
Thematic analysis	Recurring ideas, values, and meanings	What broader ideas does the narrative communicate?	Thematic interpretation	NEF examines the narrative-world organisation through which such meanings may acquire significance rather than treating theme as its primary unit of analysis.
Moral analysis	Ethical lessons, values, and normative positions	What lesson or value does the narrative communicate?	Moral interpretation	NEF does not assume a single moral lesson; it examines how agency, relationships, practices, situations, outcomes, and representation are organised.
Ideological analysis	Social assumptions, values, power relations, and systems of meaning	What social or ideological assumptions are represented or reproduced?	Ideological interpretation	NEF can provide a narrative-world description that may subsequently be examined through ideological analysis, but it does not reduce configuration to ideology.
Reader-oriented analysis	Reader interpretation, response, and meaning construction	How does a reader construct meaning from the narrative?	Reader interpretation or response	NEF primarily analyses narrative-world organisation; reader-level consequences constitute a separate level requiring empirical investigation.
Narrative Ecological Framework	Integrated organisation and interaction of six narrative-world dimensions	How are agency, relationships, practices, situations, outcomes, and representation organised and interconnected?	Narrative Ecological Profile and Narrative Ecological Configuration (NEC)	Provides an additional configuration-centred analytical level rather than replacing other approaches.

The distinctive contribution of NEF therefore lies in the **level of integration at which the narrative is analysed**. A plot analysis may establish what happens; character analysis may establish how particular characters are constructed; thematic analysis may identify recurring meanings; and ideological analysis may examine the assumptions or power relations embedded in those meanings. NEF instead examines how such elements participate in an interconnected organisation of the narrative world.

For example, the significance of an action may depend on the agent's positioning, the relationship within which the action occurs, the practice through which it is enacted, the situation that makes the action consequential, the outcome that follows, and the way these developments are represented. The configuration-centred perspective therefore attends to relations among dimensions that may remain analytically separated when each narrative element is examined independently.

Table B2

Analytical Distinction Between Dimension-Level and Configuration-Level Analysis

Analytical level	Central question	Analytical product
Narrative evidence	What is represented in the narrative?	Identifiable textual or narrative evidence
Dimension-level analysis	How is each aspect of narrative-world organisation structured?	Characterisation of the six dimensions
Cross-dimensional analysis	How do the dimensions relate to one another?	Documented reinforcement, constraint, enablement, transformation, complementarity, tension, or contradiction
Narrative Ecological Profile	What integrated pattern emerges from these relations?	Coherent profile of narrative-world organisation
Narrative Ecological Configuration	What configuration characterises the narrative world?	Concise description of the integrated NEC

The analytical sequence is therefore:

Narrative World → Six Dimensions → Dimension-level tendencies → Cross-dimensional relations → Narrative Ecological Profile → Narrative Ecological Configuration

This sequence should not be interpreted as a claim that narrative dimensions themselves operate in a fixed order. Rather, it represents the analytical movement used to describe a narrative world. As specified in Appendix A, interaction analysis may require analysts to return to earlier evidence and reconsider dimension-level interpretations.

The distinction also clarifies why an NEC should not be treated as a thematic or moral label. A statement such as “the story values cooperation” may be a legitimate thematic interpretation, but an NEC would ask how cooperation is organised across agency, relationships, practices, situations, outcomes, and representation, and how those dimensions interact to produce a particular narrative-world configuration.

Accordingly, NEF's contribution is not to provide another name for theme, character, plot, or ideology. It provides an integrative analytical level through which relations among these and other narrative-world features can be described systematically.

Concluding Remark

The Narrative Ecological Framework should therefore be understood as a configuration-centred analytical framework for describing and comparing narrative-world organisation. Its immediate contribution is conceptual and methodological: it provides a vocabulary and procedure for moving from dimension-level observations to cross-dimensional configurations. Questions concerning whether recurring NECs are recognised by readers, represented cognitively, retained across experiences, or associated with particular schemas, expectations, or interpretive orientations belong to a separate reader-level and empirical domain.
