# Supplementary material for: Narrative worlds as developmental environments: a Narrative Ecological Framework for understanding story learning and interpretation
Source: Front Psychol. 2026 Oct 7;17:1934346. doi: 10.3389/fpsyg.2026.1934346 (PMC13685022; doi:10.3389/fpsyg.2026.1934346)
Supplement: Supplementary file 3 [file Data_Sheet_3.PDF]

Appendix C. Illustrative Applications of the Narrative Ecological Framework Across Narrative Traditions

The purpose of this appendix is to demonstrate how the Narrative Ecological Framework (NEF) may be applied to diverse narrative worlds using the analytical procedure described in Appendix A. The applications are illustrative rather than empirical validation. They demonstrate one possible approach to identifying dimension-level tendencies, examining cross-dimensional relations, developing a Narrative Ecological Profile, and characterising an overall Narrative Ecological Configuration (NEC).

The selected narratives represent different narrative forms and traditions, including literary realism, social realism, fables, and non-realistic or magical narratives. Their inclusion is intended to demonstrate the analytical applicability and comparative potential of NEF, rather than to evaluate literary quality, educational value, cultural representativeness, or reader effects.

C1. *After Twenty Years*

Table C1

Narrative Ecological Profile of *After Twenty Years*

Dimension	Dimension-level characterisation	Illustrative narrative evidence	Configurational significance
**Agency Organisation**	Predominantly individual agency operating within professional and personal constraints.	Bob independently returns to fulfil the agreement, while Jimmy makes an independent decision concerning how to respond to Bob's criminal status.	Individual agency is organised within competing personal and institutional obligations.
**Relational Organisation**	Interpersonal loyalty constrained by institutional responsibility.	Jimmy's friendship with Bob remains significant, but his professional relationship to Bob as a police officer creates a conflicting obligation.	Personal affiliation and institutional responsibility are placed in tension.
**Practice Organisation**	Decision-making shaped by judgement, restraint, and professional procedure.	Jimmy chooses not to make the arrest personally and instead arranges for another officer to do so.	Agency and relational obligation are enacted through a practice that preserves professional procedure while moderating personal conflict.

Dimension	Dimension-level characterisation	Illustrative narrative evidence	Configurational significance
**Situational Organisation**	A situation structured by conflict between friendship and professional duty.	Jimmy recognises Bob as a wanted criminal while encountering him as an old friend.	The situation brings personal and institutional relations into direct tension.
**Outcome Organisation**	Institutional resolution combined with interpersonal restraint.	Bob is arrested through established police procedure, while Jimmy communicates his personal reasoning through the letter.	The outcome preserves institutional accountability while transforming the immediate interpersonal conflict into a mediated resolution.
**Representation Organisation**	Focused representation of interpersonal and institutional responsibility.	The narrative concentrates on Jimmy and Bob and foregrounds their conflicting obligations.	Representation gives continuing significance to the tension between personal loyalty and institutional responsibility.

Integrated Narrative Ecological Profile

Viewed collectively, the six dimensions organise the narrative around the interaction between individual agency, interpersonal loyalty, and institutional responsibility. The relationship between Jimmy and Bob shapes the significance of Jimmy's agency, while the institutional situation constrains how that agency can be enacted. The resulting practice, delegating the arrest while explaining the decision personally, mediates the tension between relational loyalty and professional obligation. The outcome preserves institutional accountability without eliminating interpersonal significance, and the focused representation sustains this tension as the central organisational pattern of the narrative world.

Narrative Ecological Configuration

The narrative world of *After Twenty Years* may therefore be characterised as an NEC in which individual agency operates within competing relational and institutional obligations, with professional practice mediating a situation of interpersonal–institutional tension and producing an outcome that preserves institutional accountability while retaining interpersonal significance.

C2. *The Gift of the Magi*

Table C2

Narrative Ecological Profile of *The Gift of the Magi*

Dimension	Dimension-level characterisation	Illustrative narrative evidence	Configurational significance
**Agency Organisation**	Reciprocal individual agency shaped by commitment to the relationship.	Della and Jim independently make sacrifices for one another despite material limitations.	Individual agency is organised through relational commitment rather than isolated self-interest.
**Relational Organisation**	Mutual affection, commitment, and reciprocal care.	Each character prioritises the other's happiness and attempts to provide a meaningful gift.	The relationship establishes the basis for reciprocal agency and caring practice.
**Practice Organisation**	Sacrificial and caring practices directed toward the other person.	Della sells her hair to purchase Jim's gift, while Jim sells his watch to purchase Della's gift.	Caring intentions are enacted through reciprocal material sacrifice.
**Situational Organisation**	Material constraint combined with the desire to express affection.	Limited financial resources create a practical problem concerning the exchange of meaningful gifts.	Material constraint intensifies the significance of relationally motivated practices.
**Outcome Organisation**	Material outcomes are transformed into relational significance.	The gifts become unusable in their immediate material form because each character has sacrificed what the other's gift was intended for.	The apparent practical failure of the gifts is transformed into recognition of reciprocal sacrifice.
**Representation Organisation**	The narrative foregrounds reciprocal sacrifice and gives symbolic significance to the outcome.	The gifts and sacrifices are framed as evidence of the depth of the relationship.	Representation shifts significance from material utility to relational meaning.

Integrated Narrative Ecological Profile

The six dimensions are organised around reciprocal commitment under conditions of material constraint. Della and Jim's relational commitment shapes their individual agency, while their caring intentions become enacted through sacrificial practices. The resulting situation produces an apparent practical problem, but the outcome transforms that problem by revealing the reciprocal nature of their sacrifices. Representation further shifts attention from the material utility of the gifts to the significance of the relationship.

Narrative Ecological Configuration

The narrative may therefore be characterised as an NEC in which reciprocal agency operates within a mutually supportive relationship, with caring practices shaping a situation of material constraint and

producing an outcome whose significance is derived primarily from reciprocal relational commitment rather than material utility**.**

C3. *Idgah*

Table C3

Narrative Ecological Profile of *Idgah*

Dimension	Dimension-level characterisation	Illustrative narrative evidence	Configurational significance
**Agency Organisation**	Child agency shaped by practical judgement and concern for another person.	Hamid evaluates possible purchases and chooses an object that he believes will benefit his grandmother.	Individual agency is organised through practical reasoning and relational responsibility.
**Relational Organisation**	Strong attachment, care, and responsibility within a grandparent–child relationship.	Hamid's concern for his grandmother shapes his choices during the festival.	Relational commitment provides the context in which his agency acquires significance.
**Practice Organisation**	Practical decision-making and selective use of limited resources.	Hamid forgoes treats and toys and purchases tongs for his grandmother.	Practical reasoning translates relational concern into a concrete action.
**Situational Organisation**	Material limitation and competing opportunities during the festival.	Hamid has limited money while other children purchase attractive treats and toys.	Material scarcity creates a situation in which relational priorities compete with immediate personal desires.
**Outcome Organisation**	The purchase produces practical usefulness and emotional significance.	The tongs address a recurring practical difficulty for Hamid's grandmother.	The outcome validates the practical and relational reasoning underlying Hamid's choice.
**Representation Organisation**	The narrative foregrounds care, sacrifice, and practical usefulness.	Hamid's choice is contrasted with the immediate pleasures sought by other children.	Representation gives greater significance to relational care than to immediate material gratification.

Integrated Narrative Ecological Profile

The narrative organises Hamid's agency through a combination of practical reasoning and relational responsibility. His relationship with his grandmother shapes his priorities, and these priorities become enacted through a practice of selective spending under material constraint. The resulting outcome gives practical and emotional significance to his choice, while representation contrasts his decision with the immediate consumption-oriented choices of the other children.

Narrative Ecological Configuration

The narrative may therefore be characterised as an NEC in which individual agency is organised through relational responsibility, with practical and experience-based practices transforming a situation of material constraint into an outcome whose significance is defined by care and usefulness rather than immediate personal gratification**.**

C4. *The Monkey and the Crocodile*

Table C4

Narrative Ecological Profile of *The Monkey and the Crocodile*

Dimension	Dimension-level characterisation	Illustrative narrative evidence	Configurational significance
**Agency Organisation**	Adaptive agency involving rapid interpretation and strategic response.	The monkey recognises the danger created by the crocodile's intentions and devises a response that allows him to escape.	Agency becomes consequential through the capacity to reinterpret a rapidly changing situation.
**Relational Organisation**	Friendship and cooperation are undermined by deception and competing interests.	The relationship between the monkey and crocodile changes when the crocodile reveals the intention to capture the monkey.	Relational trust becomes unstable and creates the conditions for strategic response.
**Practice Organisation**	Strategic reasoning, deception, and adaptive problem-solving.	The monkey uses a rapid verbal strategy to persuade the crocodile to return him to the riverbank.	Strategic practice enables agency under relational and situational constraint.
**Situational Organisation**	A rapidly changing situation involving threat and constrained options.	The monkey discovers that the crocodile intends to capture him while he is away	The sudden change in circumstances makes adaptive interpretation central to

Dimension	Dimension-level characterisation	Illustrative narrative evidence	Configurational significance
		from safety.	subsequent action.
**Outcome Organisation**	Escape through strategic adaptation.	The crocodile returns the monkey to the riverbank, allowing the monkey to escape.	The outcome results from the interaction between situational awareness and strategic practice.
**Representation Organisation**	The narrative foregrounds intelligence and adaptive response within a changing relationship.	The contrast between physical vulnerability and strategic intelligence is emphasised.	Representation gives significance to adaptive intelligence as a means of navigating unstable circumstances.

Integrated Narrative Ecological Profile

The narrative organises agency around rapid interpretation of changing circumstances. The initially cooperative relationship becomes unstable when the crocodile's intentions change, creating a threatening situation. The monkey's strategic practice responds to this change and transforms the situation, producing an outcome in which adaptive reasoning becomes consequential. Representation foregrounds the contrast between physical vulnerability and strategic intelligence.

Narrative Ecological Configuration

The narrative may therefore be characterised as an NEC in which adaptive individual agency operates within an unstable relationship, with rapid situational interpretation enabling strategic practice and producing an outcome through which cognitive responsiveness becomes consequential.

C5. *The Lion and the Mouse*

Table C5

Narrative Ecological Profile of *The Lion and the Mouse*

Dimension	Dimension-level characterisation	Illustrative narrative evidence	Configurational significance
**Agency Organisation**	Unequal physical capacities coexist with the capacity for consequential action.	The lion possesses physical strength and power, while the mouse, despite physical vulnerability, demonstrates	The significance of agency is not determined solely by physical capacity.

Dimension	Dimension-level characterisation	Illustrative narrative evidence	Configurational significance
		initiative and effective action.	
**Relational Organisation**	An initially asymmetrical relationship develops toward reciprocity and mutual dependence.	The lion initially holds power over the mouse but later becomes dependent on the mouse's assistance.	A change in circumstances transforms the relational significance of their respective capacities.
**Practice Organisation**	Decisions and actions enact changing relational possibilities.	The lion spares the mouse, and the mouse later acts to free the lion.	Reciprocal practices transform the significance of the initial power asymmetry.
**Situational Organisation**	A later situation reverses the practical relevance of the characters' capacities.	The lion becomes trapped and requires assistance from the mouse.	The changed situation alters which character's capacities are consequential.
**Outcome Organisation**	The problematic situation is transformed through reciprocal assistance.	The mouse frees the lion, reversing the earlier dependence.	The outcome reconfigures the significance of the earlier relationship.
**Representation Organisation**	The narrative foregrounds reversal, reciprocity, and the significance of seemingly minor actions.	The mouse's small physical capacity becomes consequential in the later situation.	Representation gives continuing significance to the transformation from asymmetry to reciprocity.

Integrated Narrative Ecological Profile

The six dimensions form an interconnected configuration in which the lion's greater physical capacity establishes an initial relational asymmetry, which shapes the significance of his decision to spare the mouse. The mouse's later intervention demonstrates how that relationship can be transformed through practice when a changed situation alters the relevance of their respective capacities. The resulting outcome changes the significance of the earlier interaction, while representation foregrounds the reversal of dependence and gives it continuing significance.

Narrative Ecological Configuration

The narrative may therefore be characterised as an NEC in which initially asymmetrical agency and relational positioning are transformed through reciprocal practice when a changed situation reverses the relevance of the characters' respective capacities, producing an outcome that reconfigures the significance of their earlier relationship.

C6. *Cinderella*

Table C6

Narrative Ecological Profile of *Cinderella*

Dimension	Dimension-level characterisation	Illustrative narrative evidence	Configurational significance
**Agency Organisation**	Constrained agency combined with persistence and eventual expanded opportunity.	Cinderella remains constrained within her household circumstances but continues to seek participation in the social event.	Agency is initially restricted by relational and situational conditions and later becomes more consequential as those conditions change.
**Relational Organisation**	Hierarchical and exclusionary household relations contrasted with later recognition and affiliation.	Cinderella is subordinated by her stepfamily but subsequently receives support and recognition.	Changes in relational positioning accompany changes in agency and social possibility.
**Practice Organisation**	Persistence, assistance, preparation, and participation.	Cinderella completes household tasks, seeks assistance, prepares for the ball, and participates despite exclusion.	Practices sustain agency under constraint and become consequential when opportunities change.
**Situational Organisation**	Social exclusion followed by an opportunity for participation and transformation.	Cinderella is initially excluded from the ball but subsequently gains access to it.	A change in situation alters the possibilities available to Cinderella.
**Outcome Organisation**	Transformation of social position and relational circumstances.	Cinderella's identity is recognised and her social circumstances change.	The outcome reorganises previous relations of exclusion and recognition.
**Representation Organisation**	Contrast between exclusion and recognition, constraint and transformation.	The narrative emphasises Cinderella's movement from marginalisation toward recognition.	Representation gives significance to the transformation of social positioning.

Integrated Narrative Ecological Profile

The narrative organises constrained agency within unequal relational and situational conditions. Cinderella's persistence is enacted through practices that maintain her participation possibilities

despite exclusion. The situation changes through external intervention, enabling different forms of action and interaction. The resulting outcome transforms her social and relational position, while representation foregrounds the movement from exclusion toward recognition.

Narrative Ecological Configuration

The narrative may therefore be characterised as an NEC in which constrained agency is reorganised through changing relational and situational conditions, while transformative intervention alters the possibilities for practice and enables a shift from exclusion toward recognition and inclusion**.**

C7. *The Snake Prince*

Table C7

Narrative Ecological Profile of *The Snake Prince*

Dimension	Dimension-level characterisation	Illustrative narrative evidence	Configurational significance
**Agency Organisation**	Shared agency involving commitment, persistence, and response to changing circumstances.	The central characters act persistently to sustain their relationship and address the challenges surrounding it.	Agency is distributed across characters whose actions become consequential through their relationship.
**Relational Organisation**	Commitment, trust, and relational continuity under difficult circumstances.	The relationship is maintained despite uncertainty and external constraints.	Relational commitment provides continuity across changing situations.
**Practice Organisation**	Persistence, care, trust, and problem-solving.	Characters undertake actions intended to protect or sustain the relationship.	Practices translate relational commitment into sustained action.
**Situational Organisation**	Magical and social circumstances that disrupt ordinary expectations.	Transformative circumstances create uncertainty concerning identity, relationship, and social position.	Changing situations require characters to adapt their practices and relational responses.
**Outcome Organisation**	Transformation and restoration of identity and relationship.	The narrative resolves the central uncertainty through a transformation of the prince and the relationship.	The outcome reorganises earlier tensions surrounding identity and belonging.
**Representation**	Symbolic representation of transformation, commitment,	Magical transformation is used to represent changes in	Representation connects external transformation with

Dimension	Dimension-level characterisation	Illustrative narrative evidence	Configurational significance
**Organisation**	and relational continuity.	identity and relationship.	the significance of relational commitment.

Integrated Narrative Ecological Profile

The six dimensions are organised around shared agency and relational commitment under changing magical and social conditions. The characters' practices sustain the relationship while responding to situations that disrupt ordinary expectations. The eventual outcome transforms the conditions surrounding identity and belonging, while representation connects this transformation to the continuing significance of relational commitment.

Narrative Ecological Configuration

The narrative may therefore be characterised as an NEC in which shared agency and relational commitment operate within changing magical and social conditions, with sustained practices of trust and persistence interacting with transformative situations to produce an outcome that reorganises identity, relationship, and social belonging**.**

C8. Cross-Narrative Comparison

Table C8

Comparative Summary of Narrative Ecological Profiles and Configurations Across Narrative Traditions

Story	Narrative form	Dominant Narrative Ecological Profile	Overall Narrative Ecological Configuration
After Twenty Years	Literary realism	Individual agency, institutional responsibility, interpersonal loyalty, professional judgement	Individual agency operates within competing relational and institutional obligations, with professional practice mediating interpersonal–institutional tension.
The Gift of the Magi	Literary realism	Reciprocal agency, relational commitment, caring practice, material constraint	Reciprocal agency operates within a mutually supportive relationship, with caring practices transforming material constraint into relational significance.

Story	Narrative form	Dominant Narrative Ecological Profile	Overall Narrative Ecological Configuration
Idgah	Social realism	Practical agency, relational responsibility, resource constraint, care-oriented practice	Individual agency is organised through relational responsibility, with practical reasoning transforming material constraint into an outcome defined by care and usefulness.
The Monkey and the Crocodile	Fable	Adaptive agency, unstable relational organisation, strategic practice, situational responsiveness	Adaptive agency operates within an unstable relationship, with rapid situational interpretation enabling strategic practice and escape.
The Lion and the Mouse	Fable	Unequal agency, changing relational dependence, reciprocal practice, situational reversal	Initial relational asymmetry is transformed through reciprocal practice when a changed situation reverses the relevance of the characters' capacities.
Cinderella	Fairy tale	Constrained agency, hierarchical relations, persistence, situational transformation	Constrained agency is reorganised through changing relational and situational conditions, producing movement from exclusion toward recognition.
The Snake Prince	Folkloric/magical narrative	Shared agency, relational commitment, persistence, transformative situations	Shared agency and relational commitment operate within changing magical and social conditions, producing transformation in identity, relationship, and belonging.

Cross-Narrative Interpretation

The illustrative applications demonstrate that the six dimensions can be used to describe and compare narrative worlds across different narrative forms and traditions. The analyses do not depend on a single genre, cultural setting, or representational mode. Instead, each narrative can be examined through the same six analytical dimensions, followed by consideration of the relations among those dimensions and synthesis into a Narrative Ecological Profile and overall Narrative Ecological Configuration.

The comparison also demonstrates why configuration-level analysis is distinct from identifying individual narrative features. Narratives may share particular descriptors while differing in how those descriptors are organised and related. Conversely, narratives that differ in plot, setting, characters, or representational mode may exhibit comparable organisational relations among their dimensions. The relevant basis of comparison is therefore not descriptor overlap alone but the organisation of dimensions and the relations among them.

For example, *The Monkey and the Crocodile* and *The Lion and the Mouse* are both fables involving anthropomorphic animals, yet their configurations differ substantially. The former organises agency and practice around rapid situational adaptation within an unstable relationship, whereas the latter organises agency through complementary contributions and changing relational dependence. Similarly, the realistic narratives differ in how individual agency, relationships, practices, situations, outcomes, and representation are organised despite their shared realistic mode.

These comparisons illustrate the analytical value of the configuration-centred perspective proposed by NEF. The framework makes it possible to move from identifying narrative features to describing how multiple dimensions are organised together and how their relations shape the structure and significance of the narrative world.

The applications presented here remain illustrative. They do not establish that readers recognise the identified configurations, extract corresponding patterns, develop schemas from them, generalise them across experiences, or adopt particular interpretive orientations. Such questions concern reader-level processes and require empirical investigation involving readers and appropriate measures. The present appendix therefore provides evidence of the applicability and descriptive potential of NEF as a qualitative framework, rather than evidence of developmental or psychological effects.
